# Zinc finger protein ZFP36L1 inhibits influenza A virus through translational repression by targeting HA, M and NS RNA transcripts

**DOI:** 10.1093/nar/gkaa458

**Published:** 2020-06-18

**Authors:** Ren-Jye Lin, Chih-Heng Huang, Ping-Cheng Liu, I-Chieh Lin, Yu-Ling Huang, An-Yu Chen, Hsin-Ping Chiu, Shin-Ru Shih, Li-Hsiung Lin, Shu-Pei Lien, Li-Chen Yen, Ching-Len Liao

**Affiliations:** Institutional affiliations: 1National Mosquito-Borne Diseases Control Research Center, National Health Research Institute, Miaoli, Taiwan; Department of Microbiology and Immunology, National Defense Medical Center, Taipei, Taiwan; Ph.D. Program in Medical Biotechnology, Taipei Medical University, Taipei, Taiwan; Department of Microbiology and Immunology, National Defense Medical Center, Taipei, Taiwan; Institute of Preventive Medicine, National Defense Medical Center, New Taipei, Taiwan; Department of Microbiology and Immunology, National Defense Medical Center, Taipei, Taiwan; Institute of Preventive Medicine, National Defense Medical Center, New Taipei, Taiwan; National institute of Infectious Diseases and Vaccinology, National Health Research Institutes, Miaoli, Taiwan; Institute of Preventive Medicine, National Defense Medical Center, New Taipei, Taiwan; Department of Medical Biotechnology and Laboratory Science, College of Medicine, Chang Gung University, Taoyuan, Taiwan; Research Center for Emerging Viral Infections, College of Medicine, Chang Gung University, Taoyuan, Taiwan; Department of Medical Biotechnology and Laboratory Science, College of Medicine, Chang Gung University, Taoyuan, Taiwan; Research Center for Emerging Viral Infections, College of Medicine, Chang Gung University, Taoyuan, Taiwan; Institutional affiliations: 1National Mosquito-Borne Diseases Control Research Center, National Health Research Institute, Miaoli, Taiwan; National institute of Infectious Diseases and Vaccinology, National Health Research Institutes, Miaoli, Taiwan; Department of Microbiology and Immunology, National Defense Medical Center, Taipei, Taiwan; Institutional affiliations: 1National Mosquito-Borne Diseases Control Research Center, National Health Research Institute, Miaoli, Taiwan; Department of Microbiology and Immunology, National Defense Medical Center, Taipei, Taiwan; National institute of Infectious Diseases and Vaccinology, National Health Research Institutes, Miaoli, Taiwan; Graduate Institute of Life Sciences, National Defense Medical Center, Taipei, Taiwan

## Abstract

ZFP36L1, a CCCH-type zinc finger protein, is an RNA-binding protein that participates in controlling cellular mRNA abundance and turnover by posttranscriptional regulation. Here, we demonstrated that ZFP36L1 has an important role in host defense against influenza A virus (IAV) infection. Overexpression of ZFP36L1 reduced IAV replication via translational repression of HA, M and NS RNA segment transcripts. IAV infection upregulated cellular ZFP36L1 expression, and endogenous ZFP36L1 knockdown significantly enhanced IAV replication. ZFP36L1 directly binds to IAV NS1 mRNA in the cytoplasm and blocks the expression and function of NS1 protein. Mutation of CCCH-type zinc finger domains of ZFP36L1 lost its antiviral potential and NS1 mRNA binding. Thus, ZFP36L1 can act as a host innate defense by targeting HA, M and NS mRNA transcripts to suppress viral protein translation.

## INTRODUCTION

Control of posttranscriptional RNA regulation via cellular mRNA decay and translation inhibition mechanisms plays an important role in the host defense against RNA virus infection. Recently, growing evidence indicates that RNA binding protein-mediated mRNA decay and translational repression machinery by CCCH-type zinc finger (ZF) proteins can function as a host innate defense against virus infection. Several of these CCCH-type ZF proteins identified as RNA-binding proteins involved in host antiviral defense by diverse antiviral mechanisms are tristetraprolin (TTP) (1), monocyte chemotactic protein-induced protein-1 (MCPIP1) (2–4), zinc-finger antiviral protein (ZAP) (5), target of Egr1 (TOE1) (6), tetrachlorodibenzo-p-dioxin (TCDD)-inducible poly (ADP-ribose) polymerase (TIPARP/PARP7) (7) and long isoform of PARP12 poly(ADP-ribose) polymerase 12 (PARP12/ZC3H1) (8). TTP itself inhibits HIV-1 production by directly binding to genomic RNA and enhancing HIV RNA transcript splicing. MCPIP1 has broad-spectrum antiviral effects against Japanese encephalitis virus (JEV) (2), dengue virus (DENV) (2), hepatitis C virus (HCV) (3) and HIV (4) via viral RNA binding and degradation by its own RNase activity. CCCH-type ZAP exhibits antiviral activity by preventing the accumulation of viral mRNA via directly binding to viral RNA and recruiting 3′-5′ exoribonuclease exosome complex for further degradation (9,10) as well as translational repression of viral mRNA by interrupting the interactions of translational initiation factors eIF4A and eIF4G (11). TOE1, a downstream target of the immediate early gene Egr1, functions as an inhibitor of HIV-1 replication by specifically binding to a HIV-1 transactivator response element and inhibiting its activity (6). TIPARP, a ZAP-like protein, binds to sindbis virus (SINV) RNA via its ZF domain and recruits an exosome to promote the degradation of viral RNA (7). PARP12L, an interferon-stimulated gene, functions as a host defense against infection with Venezuelan equine encephalitis virus (VEE) by downregulating cellular translation (8).

The human ZF protein 36, CCCH type-like 1 (ZFP36L1, also known as TIS11b or BRF1) belongs to the ZFP36 family (also named as TIS11or TTP family) that contains two other members, ZFP36 (TTP, TIS11) and ZFP36L2 (also known as TIS11D or BRF2) (12,13). The human ZFP36 family proteins contain two tandem-repeat and highly conserved ZF domains and are generally considered RNA-binding proteins, which bind to adenine uridine (AU) rich elements (AREs) in the 3′ untranslated region (3′UTR) of mRNAs and destabilize transcripts (13–16). ZFP36L1 can regulate the abundance of certain mRNAs, such as tumor necrosis factor (TNF) (17), granulocyte-macrophage colony-stimulating factor (GM-CSF) (18), vascular endothelial growth factor (VEGF) (19,20), low-density lipoprotein (LDL) receptor (21), bcl-2 (22), and cholesterol 7α-hydroxylase (Cyp7a1) (23). Besides regulating cellular mRNA decay, ZFP36L1, as a negative regulator of *Vegf-a* gene activity during development, can downregulate the protein level of VEGF probably via translational repression (24). Thus, ZFP36L1 can negatively regulate cellular gene expression at the post-transcriptional level via mRNA decay and possibly via translational repression.

The human ZFP36 family proteins interact with cellular mRNA decay enzymes such as decapping enzymes (DCP1 and DCP2), deadenylase (CCR4-NOT-deadenylase complex), exoribonuclease (5′-3′ exoribonuclease XRN1 and 3′-5′ exoribonuclease exosome complex), which converge to promote the destabilization and degradation of mRNA by diverse pathways (25). TTP-mediated mRNA decay is initiated by binding to the ARE-containing mRNAs and promotes poly(A)-tail shortening by deadenylase enzyme (CCR4-NOT-deadenylase complex) (25,26). Subsequently, mRNA degradation can occur in the 3′-5′ RNA decay pathway by the 3′-5′ exoribonuclease exosome complex for degrading deadenylated mRNA (27). In addition to the 3′-5′ RNA decay pathway, the 5′-3′ RNA decay pathway occurs via removal of the 5′-cap structure by decapping enzymes (DCP1/DCP2), thereby promoting the sequential process of cellular 5′-3′ exoribonuclease XRN1 (25,28).

Influenza A virus (IAV) is a segmented, negative-sense single-stranded RNA virus in the family *Orthomyxoviridae*. The IAV genome possesses eight RNA segments that are encapsidated in the form of ribonucleoprotein (RNP) complexes and encode at least 16 viral proteins (29). There are nine structural proteins: the two major surface glycoproteins, hemagglutinin (HA) and neuraminidase (NA), are the receptor-binding protein and glycoside hydrolase enzyme, respectively, which mainly mediate virus entry and mature-virus particle release, respectively. M2, a proton-selective channel protein represents a minor amount on the virion surface. The major structural protein matrix protein (M1) lies beneath the viral envelope. The polymerase basic proteins 1 and 2 (PB1 and PB2), polymerase acidic protein (PA) and nucleoprotein (NP) form a viral ribonucleoprotein (vRNP) complex for viral transcription and replication (30,31). Nuclear export protein/nonstructural protein 2 (NEP/NS2) and M1 are involved in vRNPs nuclear export during the viral life cycle (32,33). Nonstructural proteins such as nonstructural protein 1 (NS1) or PB1-F2 play important roles in influencing host immune responses by interferon (IFN)-antagonist activities to expedite efficient viral replication (34,35).

Here we examined whether human ZFP36L1 had potent antiviral activity against IAV by blocking the translational process of IAV mRNA, mainly the NS, HA and M RNA segments.

## MATERIALS AND METHODS

### Virus, cell lines, chemicals and antibodies

The influenza A/WSN/33 (H1N1) virus (WSN) used in this study was propagated in Madin–Darby canine kidney (MDCK) cells, which were grown in Dulbecco's modified Eagle's medium (DMEM; Gibico) containing 10% fetal bovine serum (FBS). Viral titration was by plaque-formation assays on MDCK cells. The human lung epithelial carcinoma cell line A549 was maintained in F-12 medium (Invitrogen) supplemented with 10% FBS. Human embryonic kidney 293T (HEK293T) cells were grown in DMEM (Sigma) containing 10% FBS. The tetracycline-regulated HEK293 cell line (T-REx-293; Invitrogen) expressing HA-tagged ZFP36L1 was established as described (36) and grown in DMEM (Sigma) containing 10% tetracycline-free FBS plus 5 μg/ml blasticidin (InvivoGen) and 250 μg/ml hygromycin (InvivoGen). Doxycycline (Dox) was from Clontech. MG132 was from Merck. Polyinosine-polycytidylic acid (Poly[IC]) low molecular weight was from InvivoGen. Rabbit and mouse monoclonal anti-HA antibodies, rabbit anti-BRF1/2 (ZFP36L1) and rabbit anti-CNOT6 were from Cell Signaling. Rabbit anti-exosome C5 antibody was from Abcam. Rabbit anti-XRN1 antibody was from Novus Biologicals. Rabbit polyclonal anti-influenza A H1N1 antibodies were from Genetex. Rabbit polyclonal anti-IRF-3 antibody was from Santa Cruz Biotechnology.

### Plasmids

The cDNA fragment of human ZFP36L1 (GenBank accession no.: NM_004926) was cloned into the N-terminal HA-tagged pcDNA5/TO vector (Invitrogen) and the self-inactivating lentiviral vector (pSIN vector) as described (36). The mutant of the ZF domains (C135R and C173R) of ZFP36L1 was generated by single-primer mutagenesis (37). The eight plasmids carrying influenza A/WSN/33 virus cDNAs were a kind gift from Dr. Michael M. C. Lai (Institute of Molecular Biology, Academia Sinica, Taipei). The pSIN/NS1 plasmid carrying a silent mutation in the splice acceptor site of NS1 was generated sequentially by subcloning the cDNA of IAV NS1 into the pSIN vector. Lentivirus-based shRNA constructs (pLKO-shRNA) targeting human ZFP36L1 (TRCN0000013619), CCR4-NOT transcription complex subunit 6 (CNOT6) (TRCN00000299490), XRN1 (TRCN0000296739), exosome component 5 (ExosC5) (TRCN0000306868) and LacZ (TRCN0000072223) were from the Taiwan Nation RNAi Core Facility.

### Immunofluorescence assay

A549 cells were transduced with the lentiviral vector expressing HA-tagged ZFP36L1 protein for 72 h, then infected with IAV for 24 h. Cells were fixed with 4% formaldehyde and permeabilized in phosphate buffered saline (PBS) with 0.5% Triton X-100. IAV NP protein expression was detected with a rabbit anti-IAV NP antibody and Alexa Fluor 488 goat anti-rabbit secondary antibody (Molecular Probes). The expression of HA-tagged ZFP36L1 was detected with a mouse anti-HA antibody and Alexa Fluor 568 goat anti-mouse secondary antibody (Molecular Probes). Nuclei were stained with 4′,6′-diamidino-2-phenylindole (DAPI; Molecular Probes).

### Western blot analysis

The preparation of cell lysates, SDS-PAGE and western blot analysis were as described previously (2). Data were quantified by using ImageJ.

### Lentivirus generation and establishment of shRNA-expressing stable cell lines

The lentivirus preparation and establishment of shRNA-expressing stable cell lines were as described previously (2).

### RNA extraction, RT-PCR and real-time RT-PCR

Total cellular and viral RNA were extracted for cDNA preparation by using the RNeasy Mini Kit (Qiagen), then cDNA was synthesized by the SuperScript III First-Strand Synthesis System (Invitrogen) with oligo(dT)_20_ as a primer. For RT-PCR, oligo(dT)_20_-primed cDNA was amplified by using the specific primers for the viral mRNAs of influenza A/WSN/33 (H1N1) virus: IAV HA (5′-ATGAAGGCTTTTGTACTAGTCCTG-3′ and 5′-TCAGATGCATATTCTGCACTGC-3′), IAV NP (5′-ATGGCGACCAAAGGCAC-3′ and 5′-TTAATTGTCGTACTCCTCTGCATTG-3′), IAV M1 (5′-ATGAGTCTTCTAACCGAGGTCG-3′ and 5′-TCACTTGAATCGTTGCATCTG-3′) and IAV NS1 (5′-AGCAAAAGCAGGGTGACAAA-3′ and 5′-AGTAGAAACAAGGGTGTTTTTTATTATTA-3′), respectively. Actin mRNA level with specific primers was used as an internal control. PCR products were amplified by using Mastercycler Nexus Thermal Cyclers (Eppendorf) and analyzed by 2% agarose gel electrophoresis. For real-time RT-PCR, the cDNA was amplified by using LightCycler Fast Start DNA Master SYBR Green I on a LightCycler (Roche Diagnostics GmbH) system with the specific primers for the viral mRNAs of influenza A/WSN/33 (H1N1) virus: IAV HA (5′-GAGCTCAGTATCATCATTAGAA-3′ and 5′-TGGTCAGCTTTGGGTATGAA-3′), IAV NP (5′-GCGCCAAGCTAATAATGGTG-3′ and 5′-GGAGTGCCAGATCATCATGT-3′), IAV M1 (5′-CGGTCTCATAGGCAAATGGT-3′ and 5′-CAATATCCATGGCCTCTGCT-3′) and IAV NS1 (5′-ATTCCTTGATCGGCTTCG-3′ and 5′-GCCTGCCACTTTCTGCTT-3′), respectively. The relative viral mRNA levels were normalized to that of the GAPDH control.

### Polysome profiling

The A549 cells were transduced with the lentiviral vector expressing the HA-tagged ZFP36L1 or control LacZ proteins for 72 h, and then infected with IAV (MOI = 1) for 6 h. Cells were treated with 100 μg/ml cycloheximide (Sigma) for 5 min at 37°C, and then washed twice with ice-cold PBS containing 100 μg/ml cycloheximide. Cells were then lysed using 0.5 ml polysome lysis buffer (10 mM Tris-HCl [pH7.4], 3 mM MgCl_2_, 150 mM NaCl, 35 μg/ml digitonin and 100 μg/ml cycloheximide) supplemented with 100 U RNasin Plus inhibitor (Promega) and protease inhibitor (Thermo) on ice for 30 min. Cell debris was removed by centrifugation at 16 000 g for 7 min, and then cell extracts were loaded onto 10 ml 15–40% sucrose gradients (comprising 10 mM Tris-HCl [pH7.4], 3 mM MgCl_2_, 150 mM NaCl) and centrifuged at 39 000 rpm for 90 min in a Beckman SW41-Ti rotor at 4°C. Gradients were fractionated and the optical density at 254 nm was continuously recorded using an Isco sucrose gradient fractionation system (Teledyne Isco, Inc). Proteins from the each fraction were precipitated with trichloroacetic acid (TCA) and analyzed by western blotting using mouse monoclonal anti-40S ribosomal protein S6 (RSP6) (Invitrogen) and rabbit polyclonal anti-60S ribosomal protein L11 (RPL11) antibodies (Invitrogen). Total RNA was extracted from each fraction, and then 18S and 28S rRNA was resolved on a 1% agarose gel electrophoresis and stained with ethidium bromide.

### Isolation of nuclear and cytoplasmic fractions

IAV-infected A549 cells were fractionated into nuclear and cytoplasmic proteins by using the Nuclear/Cytosol fractionation kit (BioVision) following the manufacturer's instruction. The samples were analyzed by immunoblotting with the indicated antibodies.

### RNA immunoprecipitation (RIP) assay

A549 cells were transduced with lentiviral vectors expressing the HA-ZFP36L1 wild-type or mutant protein for 72 h, then infected with IAV (multiplicity of infection [MOI] = 0.1) for 16 h. Cell extracts were mixed with prewashed HA beads (Sigma) and incubated at 4°C overnight. Viral RNA from the complex was extracted by use of the RNeasy Total RNA kit (Qiagen). RT-PCR involved the primer pair sequences for IAV NS1 (5′-AGCAAAAGCAGGGTGACAAA-3′ and 5′-AGTAGAAACAAGGGTGTTTTTTAT TATTA-3′). The PCR product from IAV NS1 was analyzed by 2% agarose gel electrophoresis and detected by SYBR Green I staining.

## RESULTS

### Human ZFP36L1 inhibits IAV infection

**Figure 1.**
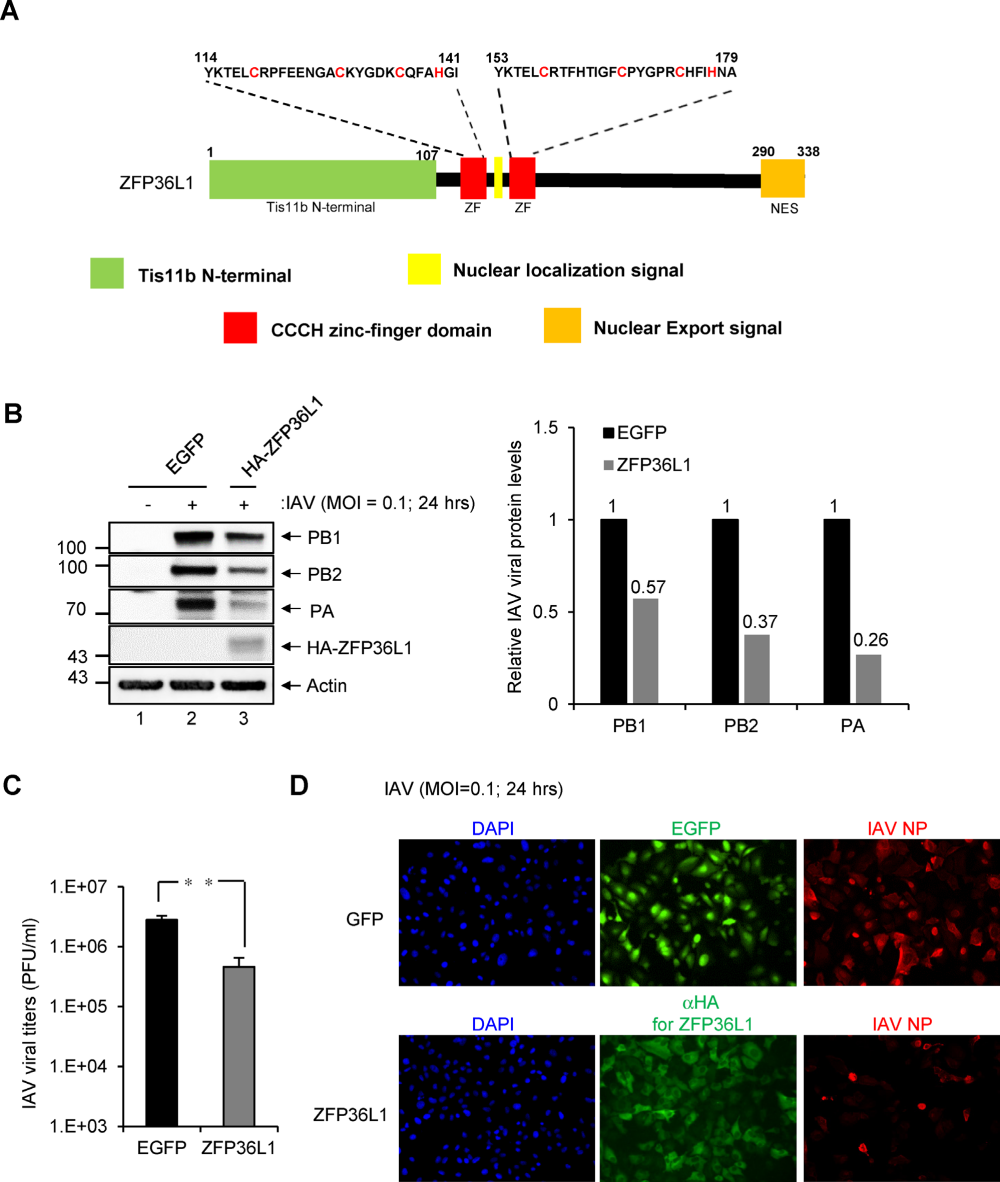
Human ZFP36L1 inhibits influenza A virus (IAV) infection. (**A**) Schematic representation of human ZFP36L1 protein containing the N-terminal domain Ti11b (Ti11b N-terminal; amino acids 1–107), two CCCH-type zinc finger (ZF) domains (amino acids 114–141 and 153–179), a putative nuclear localization signal and nuclear export signal. Shows amino acid sequence of ZF domains characterized by three conserved cysteine residues and one conserved histidine residue (red letters). (**B**) A549 cells were transduced with the lentiviral vector (multiplicity of infection [MOI] = 2) expressing HA-ZFP36L1 or control EGFP for 72 h, then infected with IAV (MOI = 0.1) for 24 h for western blot analysis of indicated IAV proteins and HA-tagged ZFP36L1, with actin as a loading control. The relative quantification of IAV proteins normalized by actin was quantified by ImageJ software. (**C**) Plaque-formation assay of infectious IAV titers (PFU/ml) in MDCK cell culture supernatants. (**D**) Indirect immunofluorescence assay of IAV NP (red), HA-tagged ZFP36L1 (green), and 4′,6′-diamidino-2-phenylindole (DAPI; blue, for nuclei). Titers of the indicated groups were compared by two-tailed Student's *t* test. Data are mean ± SD of three independent experiments. *P< 0.05; **P< 0.01; ***P< 0.001; NS: not significant.

ZFP36L1 contains two CCCH-type ZF domains characterized by three Cys residues and one His residue (Figure 1A) that can directly bind to the AREs of certain mRNAs to promote mRNA deadenylation and decay (13,14,16,25). To assess the antiviral potential of human ZFP36L1 against IAV infection, we established A549 cells overexpressing ZFP36L1 by a lentivirus expression system. Cells overexpressing ZFP36L1 or control EGFP were infected with IAV at low multiplicity of infection (MOI = 0.1). Viral load and protein expression levels were examined at 24 h post-infection (hpi). As compared with the EGFP control, overexpression of human ZFP36L1 reduced the expression of IAV proteins (PB1, PB2 and PA) as measured by western blotting in infected cells (Figure 1B). Furthermore, infectious IAV production and the expression of IAV protein NP were also significantly decreased by measurement of infectious virus titer and immunofluorescence assay, respectively (Figure 1C and D). Thus, human ZFP36L1 exhibited potent antiviral activity against IAV replication in human cells.

### ZFP36L1 inhibits the expression of IAV proteins HA, M1, M2, NS1 and NS2

**Figure 2.**
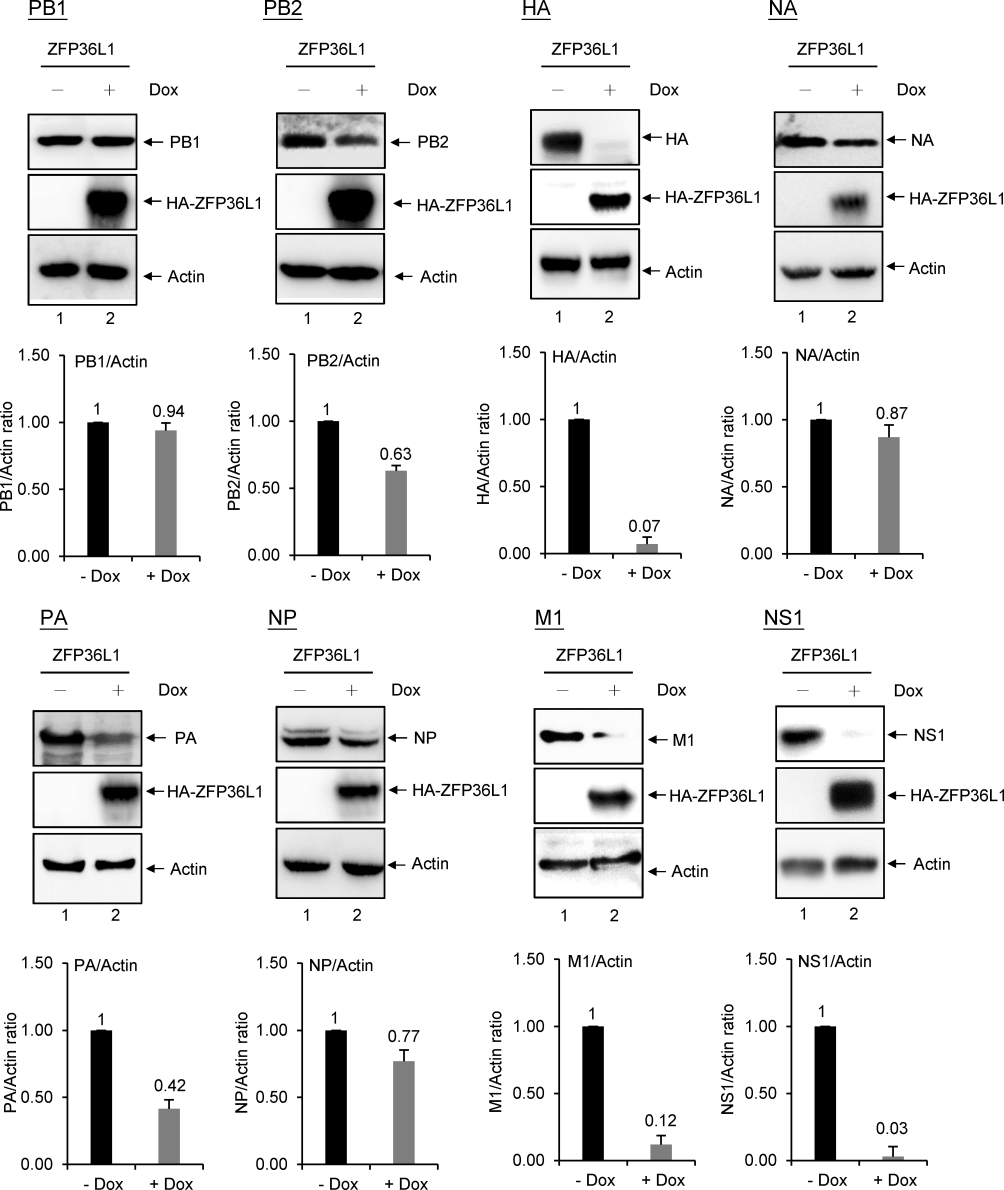
ZFP36L1 significantly reduces expression of IAV proteins, including HA, M1, M2, NS1, NS2 and PA, and to a lesser extent PA, PB2, NP and NA. Western blot analysis of protein expression in human T-REx-293 cells overexpressing ZFP36L1 induced with or without Dox (1 μg/ml) for 16 h, then transfected with plasmids expressing IAV PB1, PB2, PA, NP, HA, NA, M1 or NS1. Relative IAV protein levels normalized with actin from three independent experiments were quantified by ImageJ software.

To identify the specific targets of IAV proteins by ZFP36L1, we transfected T-REx-293 cells overexpressing ZFP36L1 induced by doxycycline (Dox) treatment with a plasmid expressing individual viral proteins. Dox-induced overexpression of ZFP36L1 greatly inhibited the protein expression of HA, M1, and NS1 and to a lesser extent PA, PB2, NP and NA (Figure 2). To further verify the ability of ZFP36L1 to downregulate the expression of these viral proteins in infected cells, ZFP36L1- or control EGFP-overexpressing A549 cells were infected with IAV (MOI = 1) at 6 hpi, and the expression of 10 viral proteins was measured by western blot analysis. Similar results were obtained: ZFP36L1-overexpressing cells mainly reduced the protein expression of HA, M1, M2, NS1 and NS2 as compared with the control EGFP (Supplementary Figure S1). These results suggest that ZFP36L1 inhibits IAV replication by blocking the expression of HA, M1, M2, NS1 and NS2 proteins derived from HA, M and NS RNA segments.

### ZFP36L1 inhibits the expression of IAV proteins HA, M1 and NS1 via translational repression

**Figure 3.**
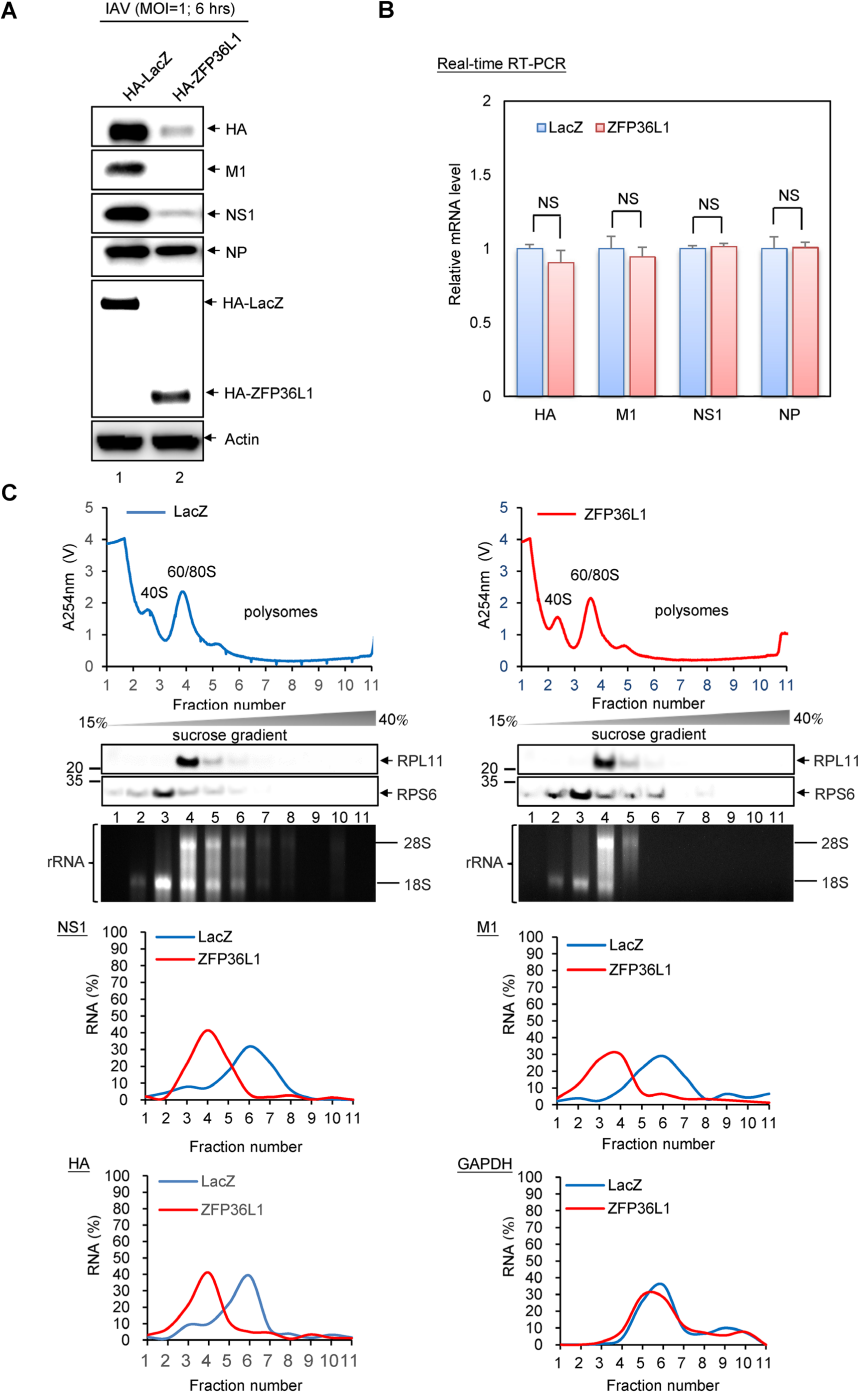
ZFP36L1 inhibits the protein but not mRNA expression of IAV M1, NS1 and HA. A549 cells were transduced with lentiviral vectors (MOI = 2) expressing HA-tagged ZFP36L1 or control LacZ. At 72 h after transduction, cells were infected with IAV (MOI = 1) for 6 h. (**A**) Western blot analysis of protein levels of IAV HA M1, NS1 and NP and actin as a loading control. (**B**) Quantitative real-time RT-PCR analysis of mRNA levels of IAV M1, NS1 and HA normalized to that of GAPDH. (**C**) Proteins and RNAs extracted from polysome fractions were collected by 10–50% sucrose gradients, respectively. Proteins from the fractions were analyzed by western blotting with the indicated antibodies. The distribution of 18S and 28S rRNA was analysed by 1% agarose gel electrophoresis and detected by ethidium bromide staining. The polysomal profiling reveals 40S, 60/80S regions, and heavy polysomal fractions (polysomes) in IAV infected cells. The mRNA level of IAV HA, NS1 and M1 from polysome fractions were analysed by quantitative real-time RT-PCR. The distribution of IAV mRNAs were represented as percentage of total mRNA (% mRNA) in the fractions. Data are mean ± SD of three independent experiments.

ZFP36L1-mediated mRNA decay is connected to the CCR4-NOT deadenylase complex as well as sequential degradation of the target mRNA via 5′-3′ exonucleolytic decay by exonuclease XRN1 and 3′-5′ exonucleolytic decay by the RNA exosome complex (13,14,25). We next examined whether the viral mRNA levels of HA, M1 and NS1 were decreased in cells with ZFP36L1 overexpression. Overexpression of ZFP36L1 in IAV-infected A549 cells blocked the expression of HA, M1, and NS1 proteins, with no change in viral mRNA expression (Figure 3A and B). Likewise, T-REx-293 cells with ZFP36L1 overexpression were transfected with a plasmid expressing HA, M1, NS1 or NP. The protein but not mRNA expression of viral HA, M1 and NS1 was greatly decreased in cells overexpressing ZFP36L1 (Supplementary Figure S2A and B). We next used polysomal profile analysis to assess ZFP36L1 role in the translation regulation of the IAV HA, M and NS mRNAs. The distribution of the IAV HA, M and NS mRNAs from polysome fractions in ZFP36L1-overexpressing cells was a tendency towards the 40S and 60/80S polysome fractions (Figure 3C), indicating that ZFP36L1 contributes to translation repression of the IAV proteins.

To further evaluate whether cellular deadenylation and RNA decay machineries participated in the anti-IAV activity of ZFP36L1, we used a lentivirus expressing shRNA-targeted CNOT6 (a component of the CCR4-NOT deadenylase complex), XRN1 or EXOSC5 (a component of the RNA exosome complex) in ZFP36L1 and EGFP-overexpressing cells. As compared with knockdown control shLacZ, knock down of CNOT6 (shCNOT6) (Supplementary Figure S3), XRN1 (shXRN1) (Supplementary Figure S4A) or EXOSC5 (shEXOSC5) (Supplementary Figure S4B) did not lose the anti-IAV effect of ZFP36L1, which suggests that deadenylation and RNA decay machineries are not involved in ZFP36L1-mediated antiviral activity against IAV.

Furthermore, to verify whether proteasomal degradation is involved in the ZFP36L1-reduced IAV protein levels, T-REx-293 cells induced to express ZFP36L1 were treated with the proteasomal inhibitor MG132 or solvent control (DMSO), then transfected with a plasmid expressing M1 or NS1. With MG132 treatment, the expression levels in M1 and NS1 protein was not rescued in cells with overexpression of ZFP36L1 as compared with DMSO treatment (Supplementary Figure S5). In contrast, MG132 treatment could stabilize and enhance the expression level of ZFP36L1, resulting in a higher reduction on the protein levels of NS1 and M1 (Supplementary Figure S5). Thus, ZFP36L1 exhibits antiviral activity against IAV via translational repression of IAV proteins.

### ZFP36L1 blocks the nuclear export of viral ribonucleoproteins (vRNPs) by blocking the expression of IAV NS2 and M1 proteins

**Figure 4.**
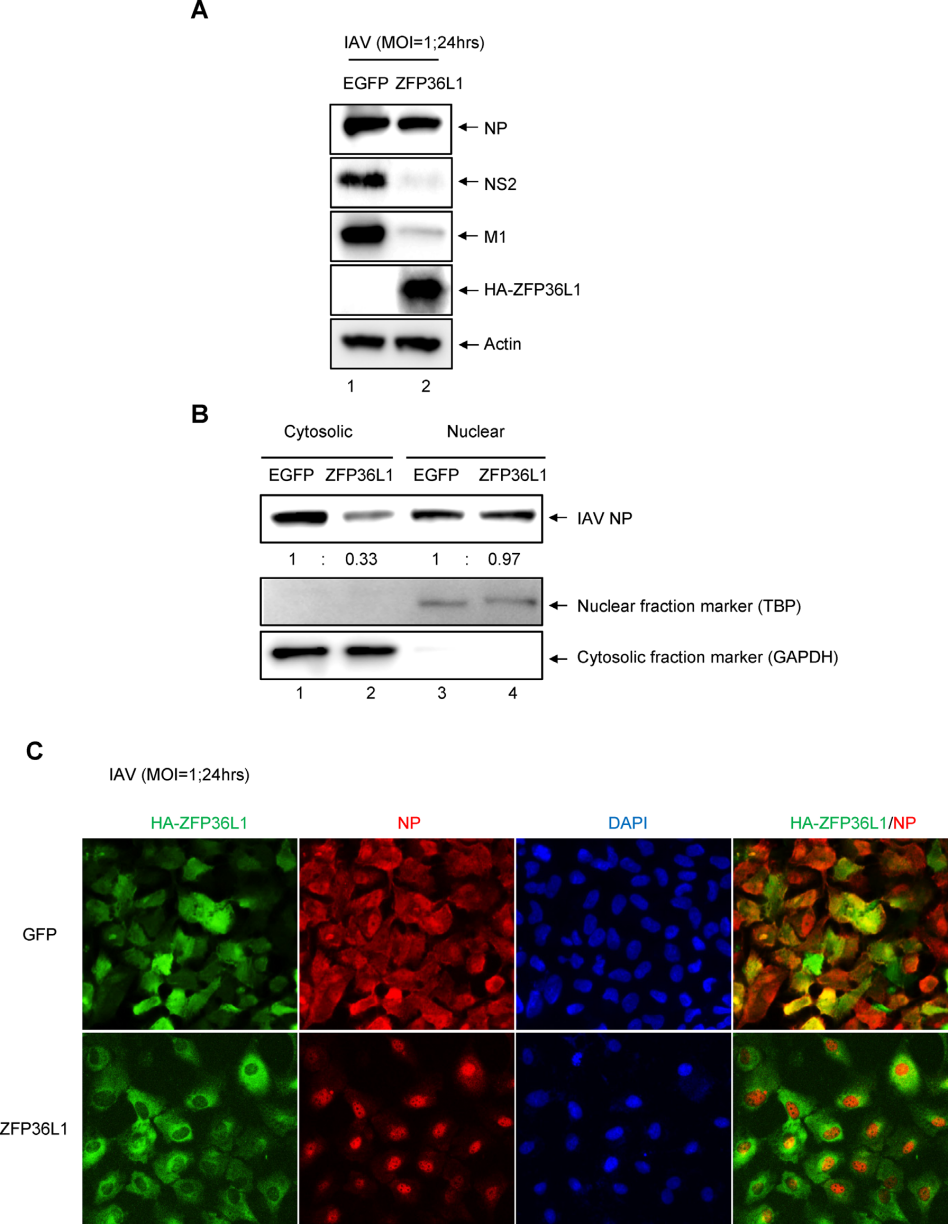
ZFP36L1 inhibits IAV NS2 and M1 expression, which blocks the nuclear export of viral ribonucleoproteins (vRNPs) in IAV-infected cells. (**A**) A549 cells were transduced with the lentiviral vector (MOI = 2) expressing HA-ZFP36L1 or control EGFP for 72 h, then infected with IAV (MOI = 1) for 24 h. Western blot analysis of protein expression of IAV NP, M1, NS2, HA-tagged ZFP36L1, and actin as a loading control. (**B**) Western blot analysis of protein level of IAV NP in nuclear and cytosolic fractions. IAV NP protein level was normalized to that of GAPDH (cytosolic fraction marker) or TATA-binding protein (TBP; nuclear fraction marker). (**C**) Cellular localization of IAV NP analyzed by indirect immunofluorescence assay. IAV NP protein, HA-ZFP36L1 protein, and nucleus were detected with anti-IAV NP Ab (red), anti-HA Ab (green), and DAPI dye (blue).

Next, we examined whether ZFP36L1 inhibits the protein levels of both NS2 and M1 to lead to retention of vRNPs in the nucleus. Cells were transduced with lentiviruses expressing ZFP3L1 or control EGFP, then infected with IAV (MOI = 1) for 24 h. As expected, the protein levels of IAV NS2 and M1 but not NP were nearly inhibited in ZFP36L1-overexpressing cells (Figure 4A). The nuclear export of vRNPs was monitored by detecting the expression of NP in the cytoplasmic fraction by nuclear/cytosolic fractionation coupled with immunoblotting analysis and the cellular localization of NP by immunofluorescence assay. By nuclear/cytosolic fractionation, immunoblotting revealed lower protein level of NP in the cytoplasm of ZFP36L1- than control EGFP-overexpressing cells (Figure 4B). Similarly, immunofluorescence assay revealed vRNPs predominantly localized in the cytoplasm in control EGFP-overexpressing cells but in the nucleus in ZFP36L1-overexpressing cells (Figure 4C). Thus, ZFP36L1 can block the expression of NS2 and M1 proteins, thereby blocking the nuclear export of vRNPs.

### CCCH-type ZF domains of ZFP36L1 are critical for its antiviral activity

**Figure 5.**
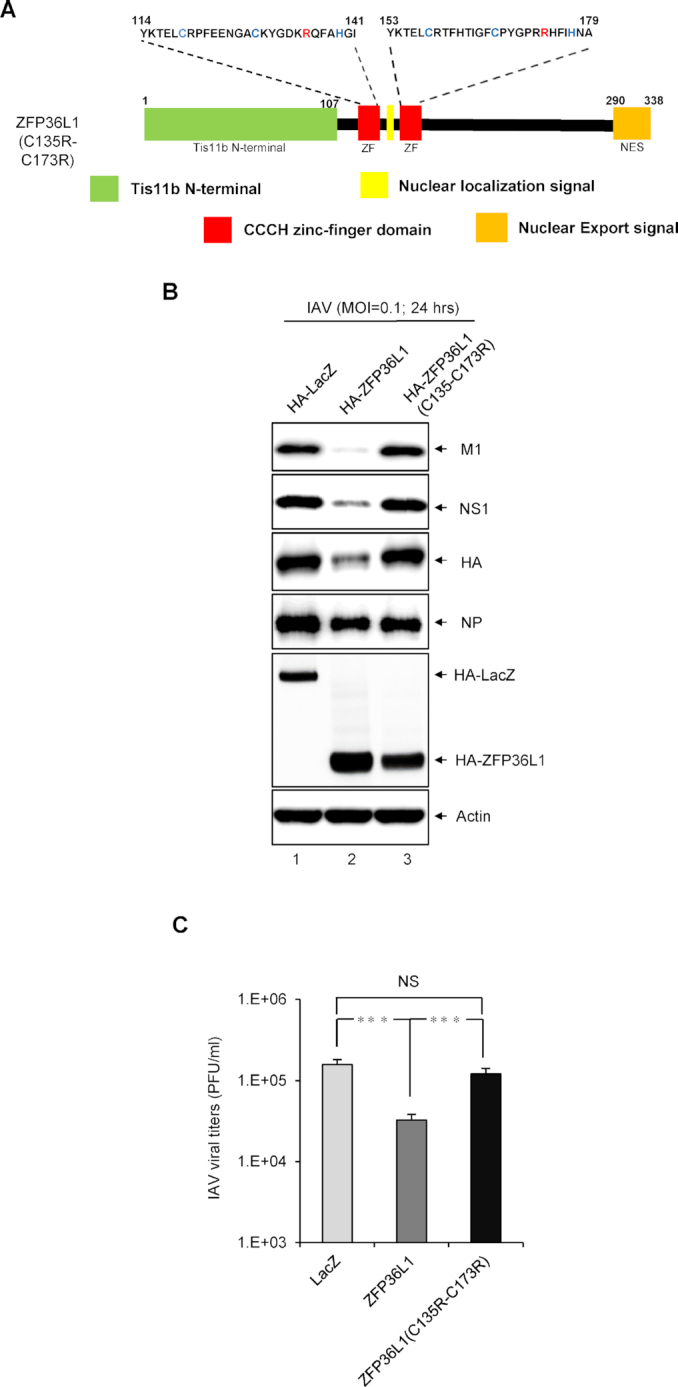
The CCCH-type ZF domains of ZFP36L1 are required for antiviral activity against IAV infection. (**A**) Schematic representation of the mutation sites (red letters) of ZF domains of human ZFP36L1 (C135R/C173R) mutant. (**B**) A549 cells were transduced with the lentiviral vector (MOI = 2) expressing HA-ZFP36L1, HA-ZFP36L1 (C135R/C173R) mutant or control EGFP for 72 h, then infected with IAV (MOI = 0.1) for 24 h. Western blot analysis of the indicated IAV proteins, HA-tagged ZFP36L1, HA-tagged LacZ, and actin as a loading control. (**C**) Plaque-formation assay of infectious IAV titers (PFU/ml) in MDCK cells. The titers of the indicated groups were compared by two-tailed Student's *t* test. Data are mean ± SD of three independent experiments. ***P< 0.001; NS: not significant.

To evaluate the importance of the ZF domains in ZFP36L1 in antiviral activity against IAV, we constructed a C135R/C173R mutant with a single point mutation at the conserved CCCH residues (ZF1:C135 and ZF2:C173) of the ZF domains (Figure 5A). As compared with ZFP36L1-overexpressing cells, cells overexpressing the ZFP36L1-C135R/C173R mutant lost nearly all inhibitory activity as determined by the protein expression of M1, NS1 and HA (Figure 5B) and viral production (Figure 5C), as did control EGFP, which suggests that the ZF domains of ZFP36L1 are required for its antiviral activity against IAV.

### ZFP36L1 interacts with NS1 mRNA via its ZF domains, which inhibits the expression and function of NS1.

**Figure 6.**
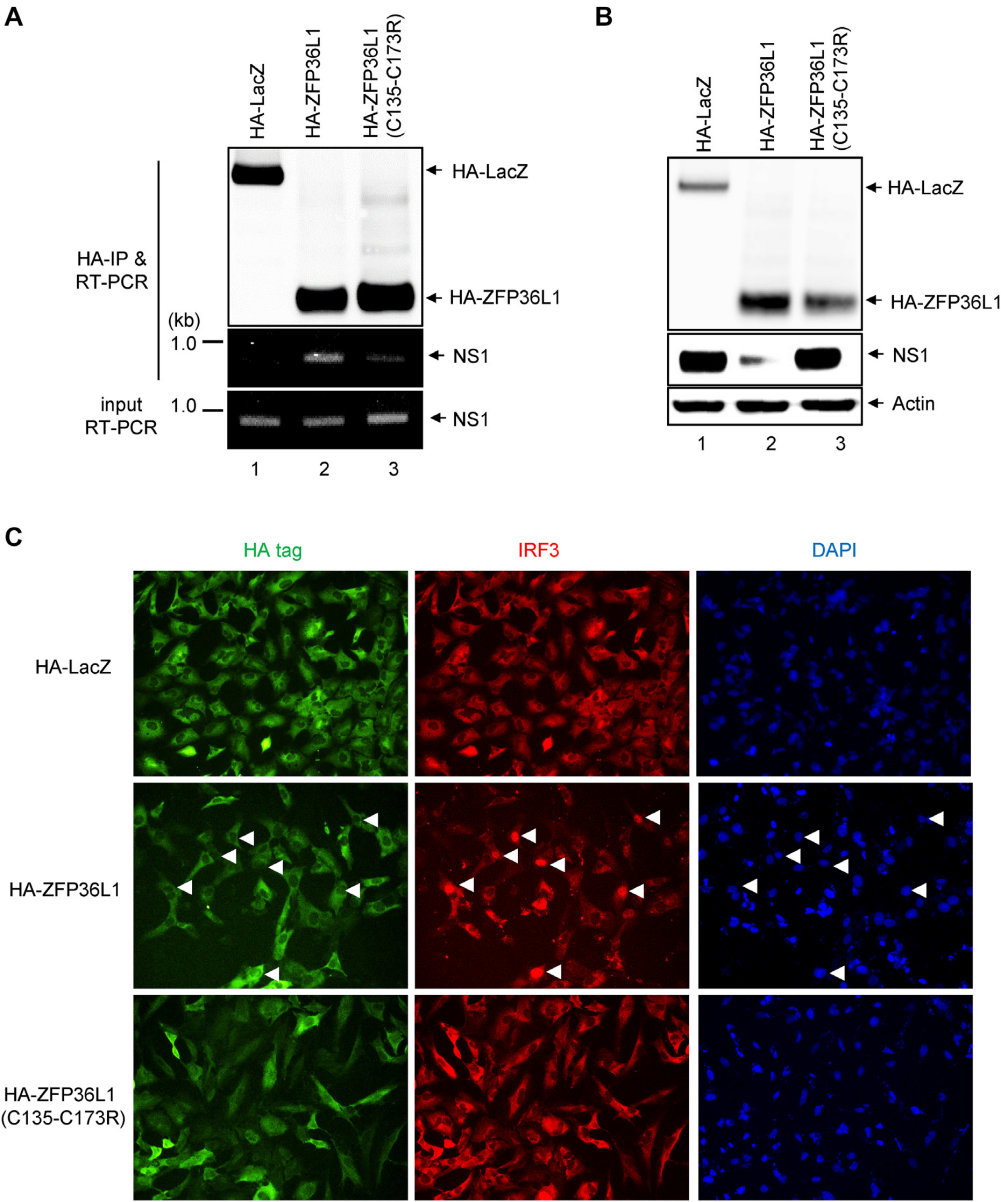
The CCCH-type ZF domains of ZFP36L1 are required for binding to NS1 mRNA and block the expression and function of NS1. (**A**) A549 cells were transduced with the lentiviral vector (MOI = 2) expressing HA-ZFP36L1 or HA-ZFP36L1 (C135R/C173R) mutant or control HA-LacZ for 72 h, then infected with IAV (MOI = 1) for 6 h. The NS1 mRNA bound with HA-tagged ZFP36L1 protein was pulled down (IP) with anti-HA affinity beads and amplified by RT-PCR with specific NS1 primers (middle panel). RT-PCR of input NS1 mRNA (lower panel) and western blot analysis of the immunoprecipitated HA-tagged LacZ and ZFP36L1 (upper panel). (**B**) A549 cells stably expressing the IAV NS1 protein by lentivirus transduction were transduced with the lentiviral vector (MOI = 2) expressing HA-ZFP36L1, HA-ZFP36L1 (C135R-C173R) mutant or control HA-LacZ for 72 h, then transfected with poly(I:C) (0.5 μg) for 24 h. (**C**) Immunofluorescence assay of the indicated HA-tagged protein (green)-, IRF3 protein (red)-, and 4′,6-diamidino-2-phenylindole (DAPI; blue)-stained cells. Arrows indicate the poly(I:C)-induced IRF3 nuclear translocation.

ZFP36L1 contains two highly conserved CCCH-type ZF domains with RNA-binding potential that directly bind to certain mRNA via ARE-dependent mechanisms (13,14,16). To verify whether ZFP36L1 is involved in viral mRNA binding and whether the CCCH-type ZF domains are essential for viral mRNA binding activity, cells were overexpressed with the HA-tagged ZFP36L1 or ZFP36L1-C135R/C173R mutant, then infected with IAV. The RNA-binding capacity of ZFP36L1 was analyzed by immunoprecipitation with an antibody against the HA tag, followed by RT-PCR for mRNA levels of HA, M1 or NS1. The mRNA of NS1 was pulled down by ZFP36L1 in IAV-infected cells (Figure 6A). ZFP36L1-C135R/C173R mutant lost its viral RNA-binding activity (Figure 6A). We further examined whether ZFP36L1 binds NS1 mRNA in the cytoplasm or nucleus. Co-localization of IAV NS1 mRNA and ZFP36L1 protein was clearly seen in the cytoplasm of IAV-infected cells (Supplementary Figure S6).

To further verify whether the expression and function of NS1 were blocked in cells with ZFP36L1 overexpression, we constructed a lentiviral vector carrying a silent mutation in the splice acceptor site of NS1 and established a stable cell line expressing NS1 by lentivirus transduction in A549 cells. ZFP36L1- but not LacZ-overexpressing or ZFP36L1-C135R/C173R mutant-overexpressing cells showed abolished NS1 expression (Figure 6B). NS1 functions as a double-stranded RNA (dsRNA) binding protein that blocks the activation of IFN regulatory factor 3 (IRF3), nuclear translocation of IRF3 and downstream induction of the type I IFN genes (38). Cells stably expressing NS1 blocked the IRF3 nuclear translocation mediated by poly IC (Supplementary Figure S7). As compared with control LacZ, ZFP36L1 overexpression blocked the nuclear translocation of IRF3, whereas overexpression of the ZFP36L1-C135R/C173R mutant lost the inhibitory effect on nuclear translocation of IRF3 (Figure 6C). These data indicate that ZFP36L1 can bind to NS1 mRNA in the cytoplasm via its ZF domains and block the expression and function of IAV NS1.

### Antiviral potential of endogenous ZFP36L1 against IAV infection

**Figure 7.**
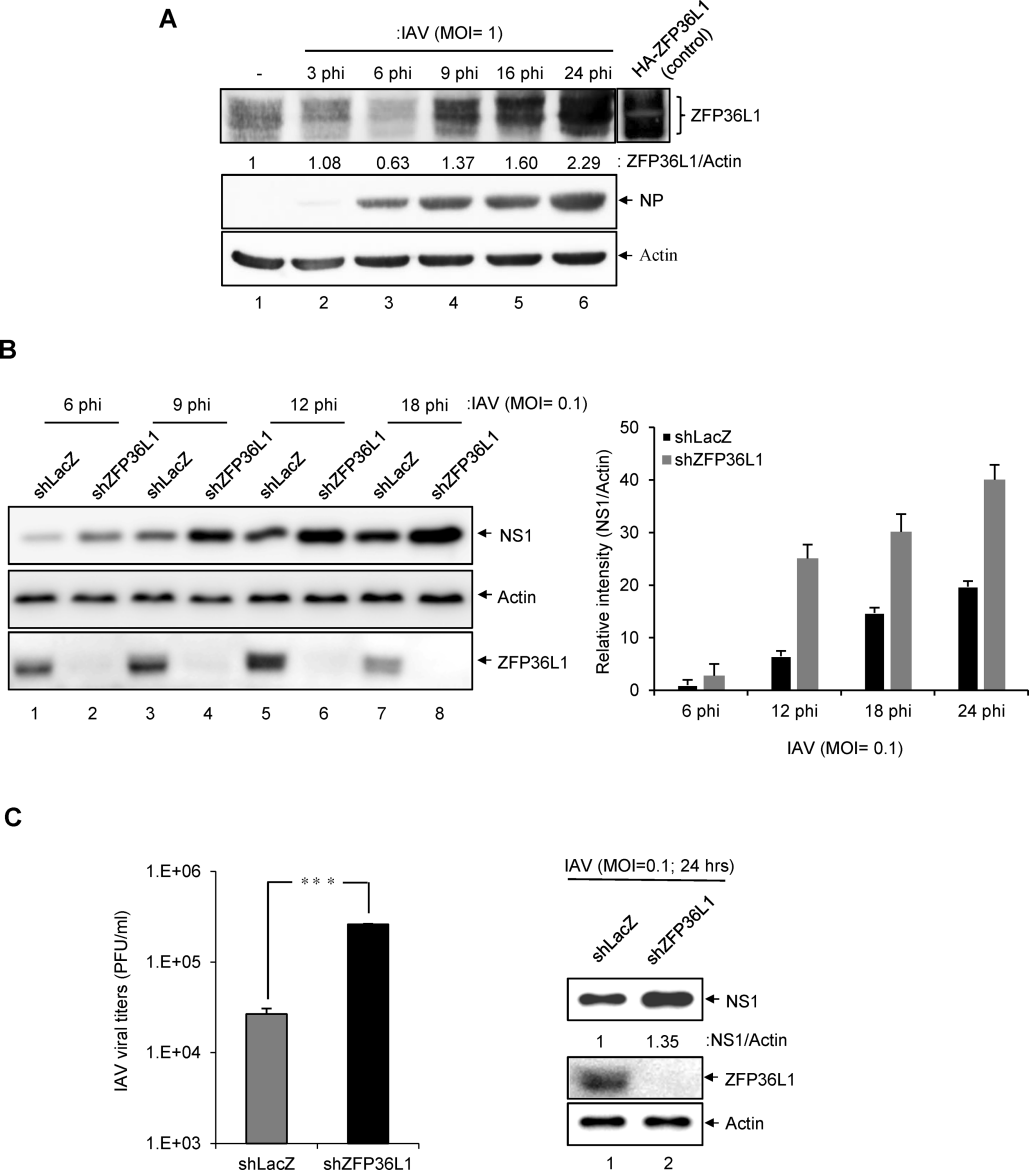
The antiviral potential of endogenous ZFP36L1 against IAV. (**A**) A549 cells were mock-infected or infected with IAV (MOI = 1) for 3, 6, 9, 12 or 24 h. Western blot analysis of protein levels of ZFP36L1, IAV NP and actin as a loading control. (**B, C**) Western blot analysis of knockdown of A549 cells stably transduced with lentivirus expressing shRNA targeting control LacZ (shLacZ) or ZFP36L1 (shZFP36L1) and infected with IAV (MOI = 0.1) for the indicated time. (**B**) Western blot analysis of protein levels of IAV NS1, ZFP36L1 and actin as a loading control. The relative quantification of IAV proteins normalized by actin was quantified by ImageJ software (**C**) Western blot analysis of protein expression of IAV NS1 at 24 h after infection and plaque-formation assay of infectious IAV titers (PFU/ml) in MDCK cells. The titers of the indicated groups were compared by two-tailed Student *t* test. Data are mean ± SD of three independent experiments. ***P< 0.001; NS: not significant.

To assess the role of endogenous ZFP36L1 in IAV infection, A549 cells were infected with IAV (MOI = 1) at various times and the protein expression of endogenous ZFP36L1 was measured. IAV-infected cells showed upregulated protein level of ZFP36L1, especially at 9, 16 and 24 hpi but to a lesser extent at 6 hpi (Figure 7A), as well as the expression level of endogenous ZFP36L1 was obviously induced by TNF-α treatment (Supplementary Figure S8). Lentivirus knockdown of endogenous ZFP36L1 (shZFP36L1) in A549 cells enhanced the protein level of NS1 at low MOI (MOI = 0.1) (Figure 7B) and high MOI (MOI = 1) (Supplementary Figure S9). Infected cells with knockdown ZFP36L1 expression showed increased NS1 protein level and IAV viral production at 24 hpi (Figure 7C). Furthermore, the rescue of the ZFP36L1 knockdown by transduction with a lentiviral vector overexpressing ZFP36L1 significantly decreased the expression of IAV protein (Supplementary Figure S10A) and infectious IAV production (Supplementary Figure S10B), respectively. These results indicate that IAV infection can induce endogenous ZFP36L1 level and knockdown of ZFP36L1 enhances viral replication, so ZFP36L1 may play a role in the host antiviral defense against IAV.

## DISCUSSION

ZFP36L1 is a family member of the CCCH-type of ZF proteins and was identified as an RNA-binding protein, which has important roles in various cellular and biological functions such as inflammation, apoptosis, proliferation, differentiation and angiogenesis (13). However, its role in the host defense response against viral infection has not been addressed. In this study, we used gene overexpression to explore the antiviral potential of human ZFP36L1 protein: ZFP36L1 overexpression could exhibit potent antiviral activity against IAV infection. Moreover, the protein level of endogenous ZFP36L1 was upregulated in response to IAV infection, and ZFP36L1 knockdown enhanced the viral replication of IAV. These findings indicate that human ZFP36L1 can function as a cellular antiviral factor to restrict viral replication in IAV-infected cells and extend the biological function of ZFP36L1 in host antiviral defense.

Post-transcriptional and -translational regulation by CCCH ZF proteins such as MCPIP1 and ZAP restrict IAV replication with distinct functions and diverse mechanisms. MCPIP1 ribonuclease, which exhibits antiviral effects via direct binding and degradation of viral RNA by itself, functionally inhibits IAV replication (2,39). The short form of ZAP (ZAPS), which lacks the C-terminal PARP domain, posttranscriptionally inhibits IAV replication at an early stage of infection and blocks the expression of PA, PB2 and NA, then reduces the encoding viral mRNA levels such as PB2 (40). The long form of ZAPL (isoforms ZAPL), with a PARP domain, has distinct antiviral activity against IAV, directly binding the IAV proteins PA and PB2 for subsequent viral protein degradation by the proteasome-dependent pathway (41). Different from the antiviral functions of MCPIP1, ZAPS and ZAPL against IAV, our data demonstrated that ZFP36L1 inhibited IAV replication by blocking protein translation without affecting viral mRNA levels (Figure 3), so the antiviral effect of ZFP36L1 participates in repressing viral mRNA translation but not viral mRNA decay. Furthermore, knockdown of CNOT6 (shCNOT6), XRN1 (shXRN1) and EXOSC5 (shEXOSC5), which are the components of cellular mRNA decay machinery recruited by ZFP36L1, did not support the antiviral effect of ZFP36L1 against IAV (Supplementary Figures S3 and S4). These findings support that the RNA decay pathway does not participate in the antiviral activity of ZFP36L1.

Recently, the ARE-binding protein ZFP36L2 was found to mediate the translational repression of pre-formed mRNA in memory T cells (42). We found that ZFP36L1 inhibited IAV replication, which mainly blocked the protein expression of HA, M1, M2, NS1 and NS2 (Supplementary Figure S1 and Figure 3) without affecting their viral mRNA expression (Figure 3). In addition, the protein but not mRNA expression of viral HA, M1, and NS1 was greatly reduced in ZFP36L1-overexpressing cells (Supplementary Figure S2). As well, the presence of MG132, a proteasome inhibitor, did not prohibit the ZFP36L1-mediated translational repression of NS1 (Supplementary Figure S6); hence, the proteasomal degradation pathway should not be involved in ZFP36L1 reducing the expression of IAV proteins. Together, our results provide strong evidence that ZFP36L1 exerts its antiviral activity against IAV via translational repression of viral mRNA transcripts.

Besides regulating mRNA stability, TTP, a ZFP36 family member, has been found to have an ARE-mRNA translational repression function (43). ZAP blocks translation of viral mRNA target by disrupting the interaction between the translational initiation factors eIF4A and eIF4G (11). Moreover, the translation of influenza virus mRNAs has been found required for the two components of the eIF4F translation initiation factor, eIF4A and eIF4G proteins (44). Thus, whether ZFP36L1 represses the translation of influenza virus mRNAs by disrupting the translational initiation factors eIF4A and eIF4G remains to be clarified.

ZFP36L1, an RNA-binding protein, contains two highly conserved CCCH-type ZF domains that are responsible for binding to AREs in the 3′ UTR of the mRNA, which leads to the instability and degradation of mRNA (14,16). The preferential binding region of ZFP36 family proteins to the consensus RNA sequence is UUAUUUAU (45–48). Mutation of the CCCH-type ZF domains of ZFP36L1 lost its antiviral activity against IAV (Figure 5B and C), so the CCCH-type ZF domains of human ZFP36L1 are essential for antiviral activity against IAV infection. Analysis of the mRNA sequence of IAV HA, M1 and NS1 revealed that these mRNAs contained a core sequence of AUUUA, a short ARE-containing region, which might be sufficient for binding ZFP36L1. Therefore, the mRNA transcripts of IAV might bind to ZFP36L1. Indeed, we found that NS1 mRNA bound to ZFP36L1 in IAV-infected cells, and mutation of the CCCH-type ZF domains of ZFP36L1 lost its RNA-binding activity and translation inhibition for NS1 mRNA (Figure 6A and B).

NS1 mRNA contains two ARE-containing regions in its 3′ UTR, ARE1 and ARE2 (Supplementary Figure S11A). To determine whether ARE regions of NS1 mRNA are critical for translation inhibition by ZFP36L1, we constructed the NS1-ARE1, -ARE2 and -ARE1/2 mutant with AUUUA mutated to AGGGA as well as 3′ UTR -truncated mutant for RNA transcripts. However, the effect of ZFP36L1 on translational repression remained unchanged (Supplementary Figure S11B), so the ARE regions of NS1 mRNA are not critical for ZFP36L1 to inhibit translation.

Besides involvement in a classical ARE-dependent pathway, TTP has been reported to downregulate the expression of mRNAs via a non-ARE-dependent pathway (49). Thus, ZFP36L1 might downregulate the translation of NS1 mRNA by binding to a non-ARE-containing target sequence. Also, ZFP36L1 might trigger a distinct mechanism to block the translation of IAV mRNA, which contributes to restrict IAV replication. More work is needed to delineate the underlying mechanism for the potent antiviral effect of ZFP36L1 on IAV.

## Supplementary Material

gkaa458_Supplemental_File
