# Supplementary material for: Zinc finger protein ZFP36L1 inhibits influenza A virus through translational repression by targeting HA, M and NS RNA transcripts
Source: Nucleic Acids Res. 2020 Jun 18;48(13):7371–84. doi: 10.1093/nar/gkaa458 (PMC7367194; doi:10.1093/nar/gkaa458)
Supplement: gkaa458_Supplemental_File [file gkaa458_supplemental_file.pdf]

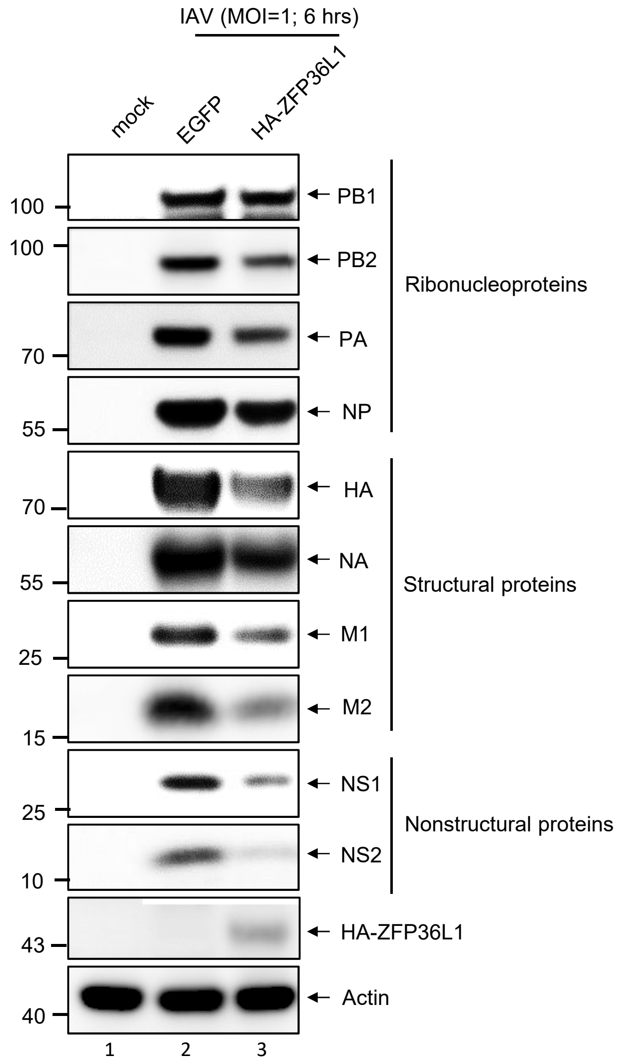
IAV (MOI=1; 6 hrs)
mock
EGFP
HA-ZFP36L1
100
PB1
100
PB2
Ribonucleoproteins
PA
70
NP
55
HA
70
NA
55
Structural proteins
M1
25
M2
15
NS1
25
Nonstructural proteins
NS2
10
HA-ZFP36L1
43
Actin
40
1
2
3

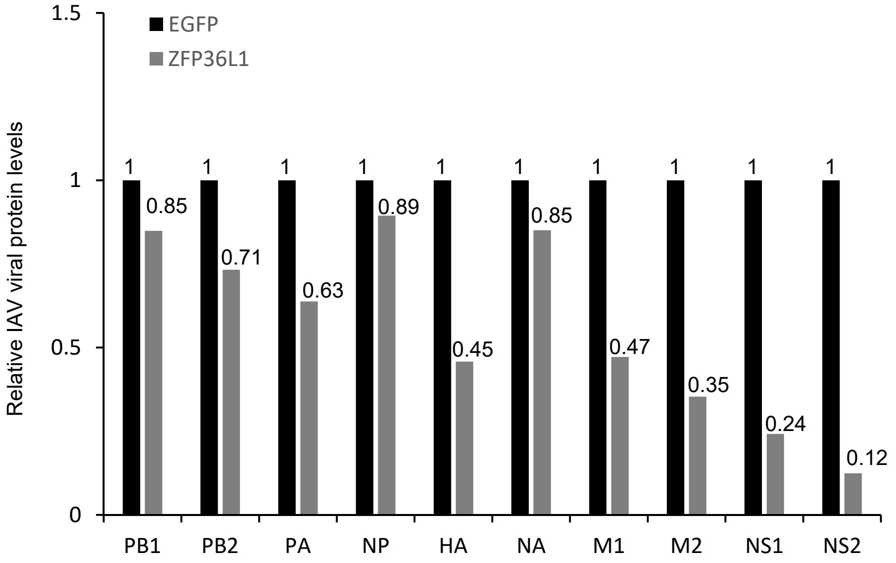
EGFP
ZFP36L1
Relative IAV viral protein levels
1.5
1
0.5
0
PB1
PB2
PA
NP
HA
NA
M1
M2
NS1
NS2
1
0.85
1
0.71
1
0.63
1
0.89
1
0.45
1
0.85
1
0.47
1
0.35
1
0.24
1
0.12

A.

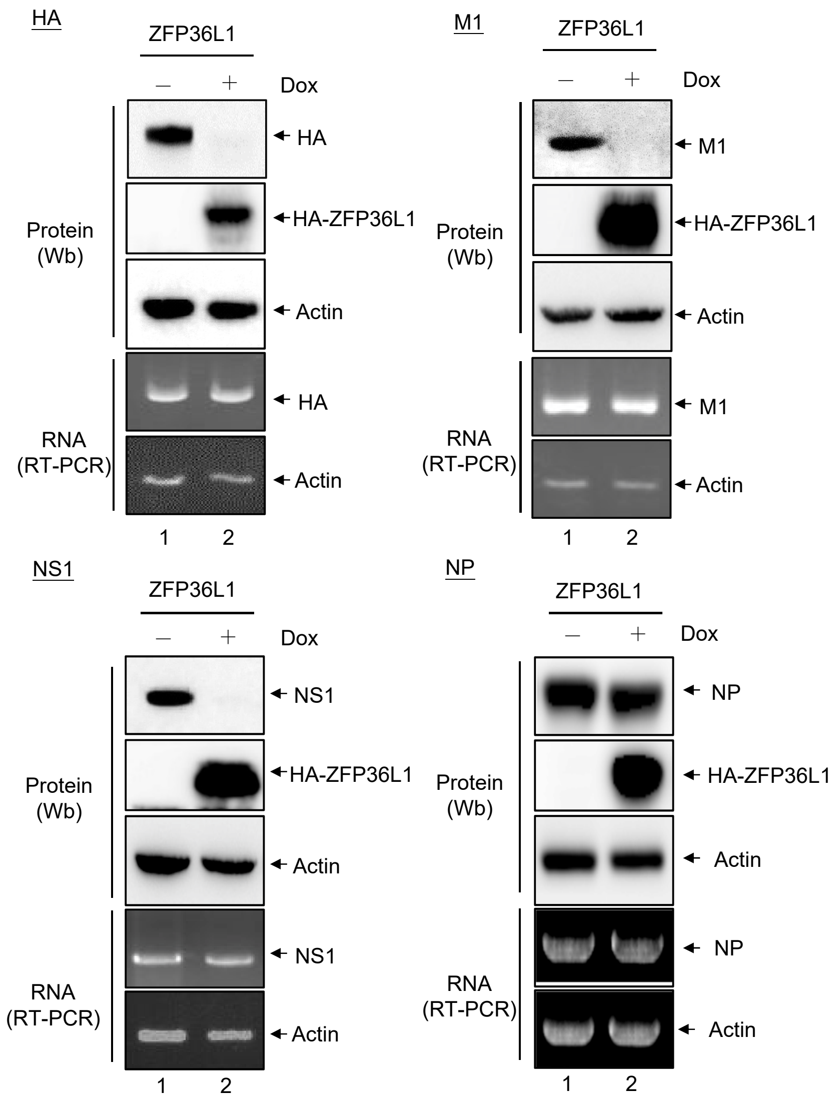
HA
ZFP36L1
– + Dox
HA
HA-ZFP36L1
Actin
Protein (Wb)
RNA (RT-PCR)
HA
Actin
1 2
M1
ZFP36L1
– + Dox
M1
HA-ZFP36L1
Actin
Protein (Wb)
RNA (RT-PCR)
M1
Actin
1 2
NS1
ZFP36L1
– + Dox
NS1
HA-ZFP36L1
Actin
Protein (Wb)
RNA (RT-PCR)
NS1
Actin
1 2
NP
ZFP36L1
– + Dox
NP
HA-ZFP36L1
Actin
Protein (Wb)
RNA (RT-PCR)
NP
Actin
1 2

B.

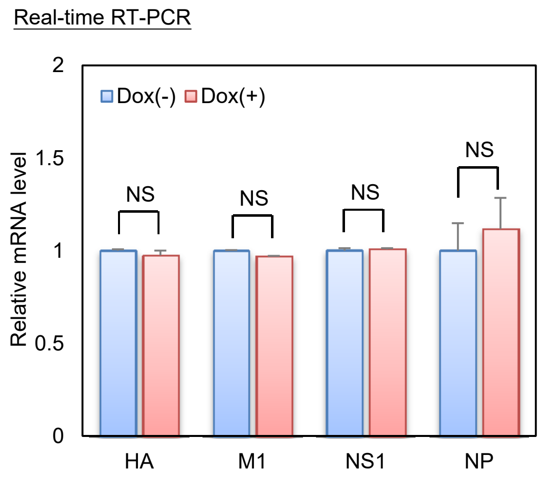
Real-time RT-PCR
Dox(-) Dox(+)
Relative mRNA level
2
1.5
1
0.5
0
NS
NS
NS
NS
HA
M1
NS1
NP

Lin *et al.,* sFig. 3

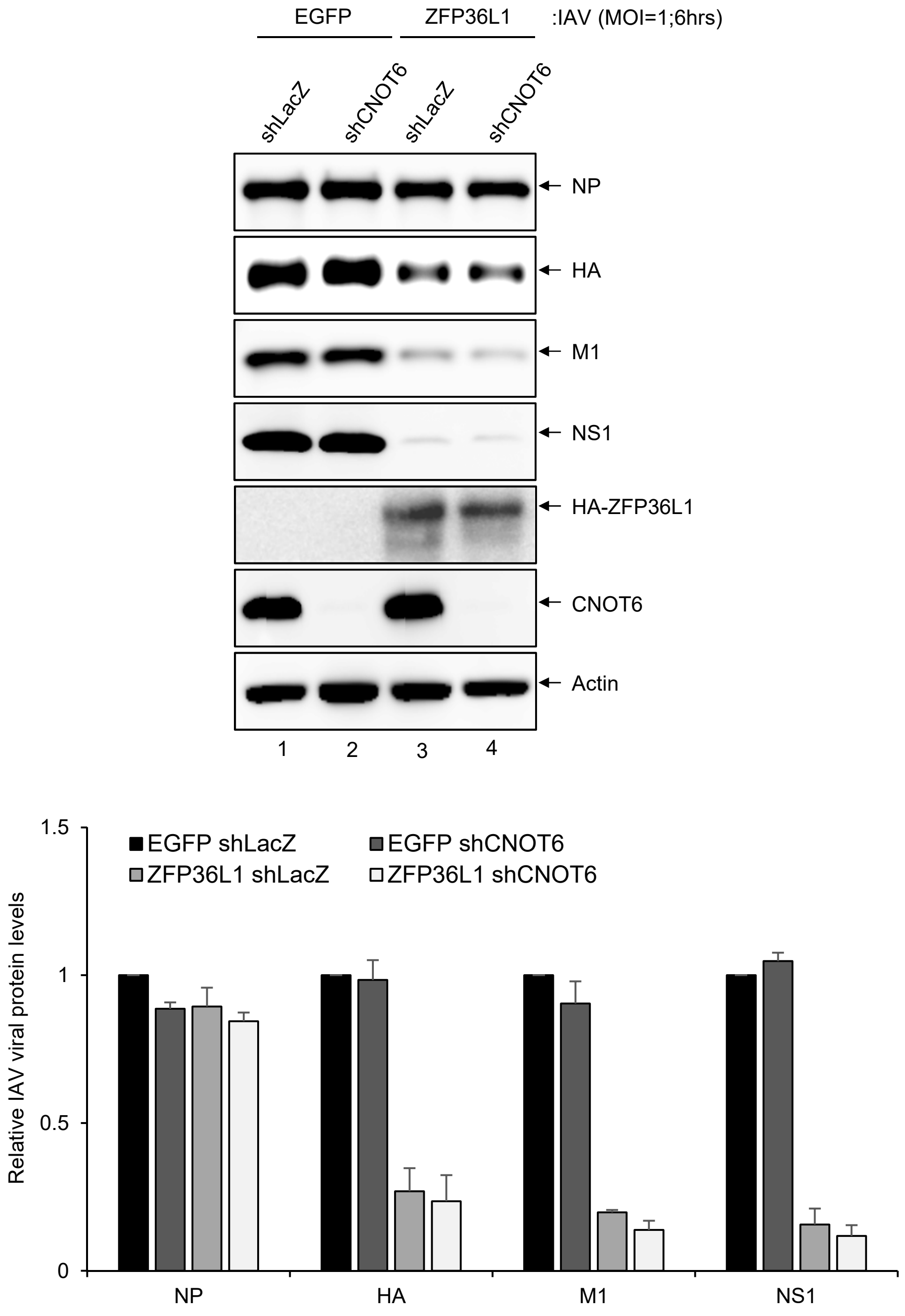

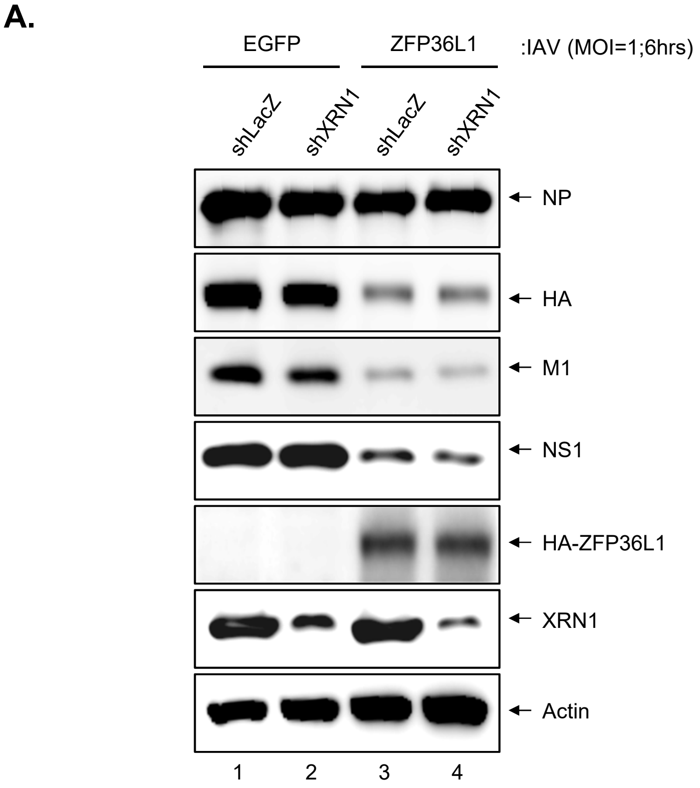
A.
EGFP
ZFP36L1
:IAV (MOI=1;6hrs)
shLacZ
shXRN1
shLacZ
shXRN1
NP
HA
M1
NS1
HA-ZFP36L1
XRN1
Actin
1
2
3
4

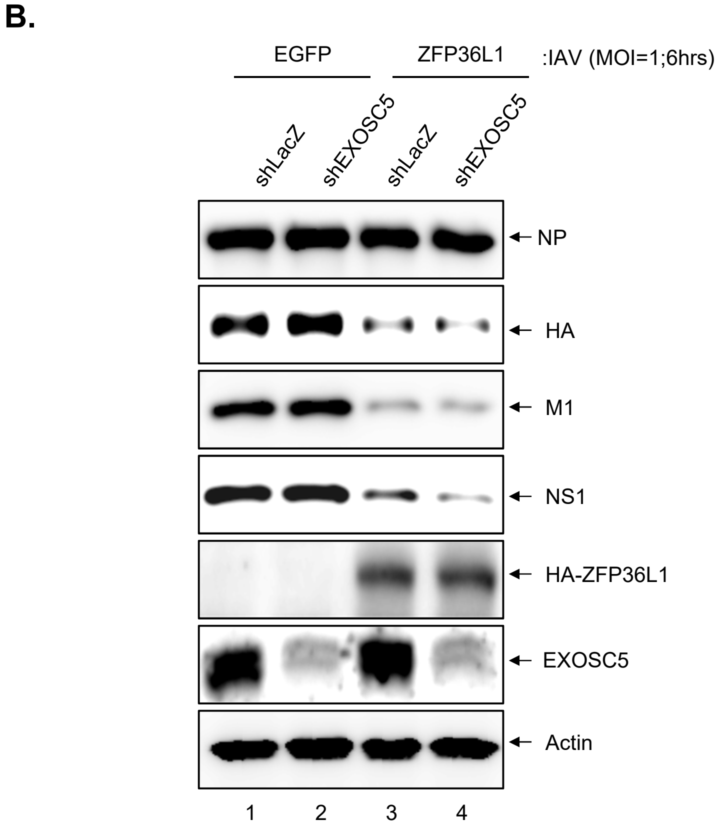
B.
EGFP
ZFP36L1
:IAV (MOI=1;6hrs)
shLacZ
shEXOSC5
shLacZ
shEXOSC5
NP
HA
M1
NS1
HA-ZFP36L1
EXOSC5
Actin
1
2
3
4

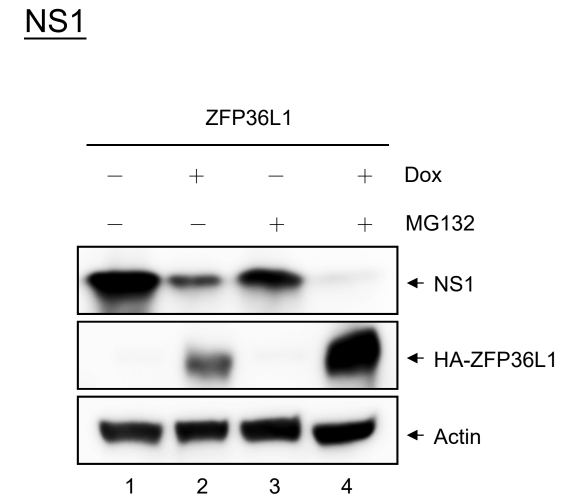
NS1
ZFP36L1
– + – + Dox
– – + + MG132
NS1
HA-ZFP36L1
Actin
1 2 3 4

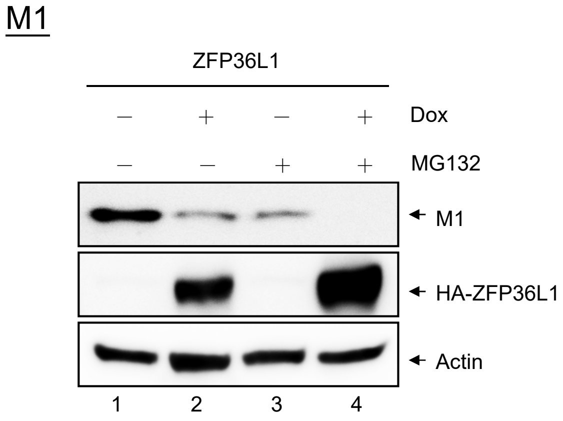
M1
ZFP36L1
– + – + Dox
– – + + MG132
M1
HA-ZFP36L1
Actin
1 2 3 4

Lin *et al.*, sFig. 6

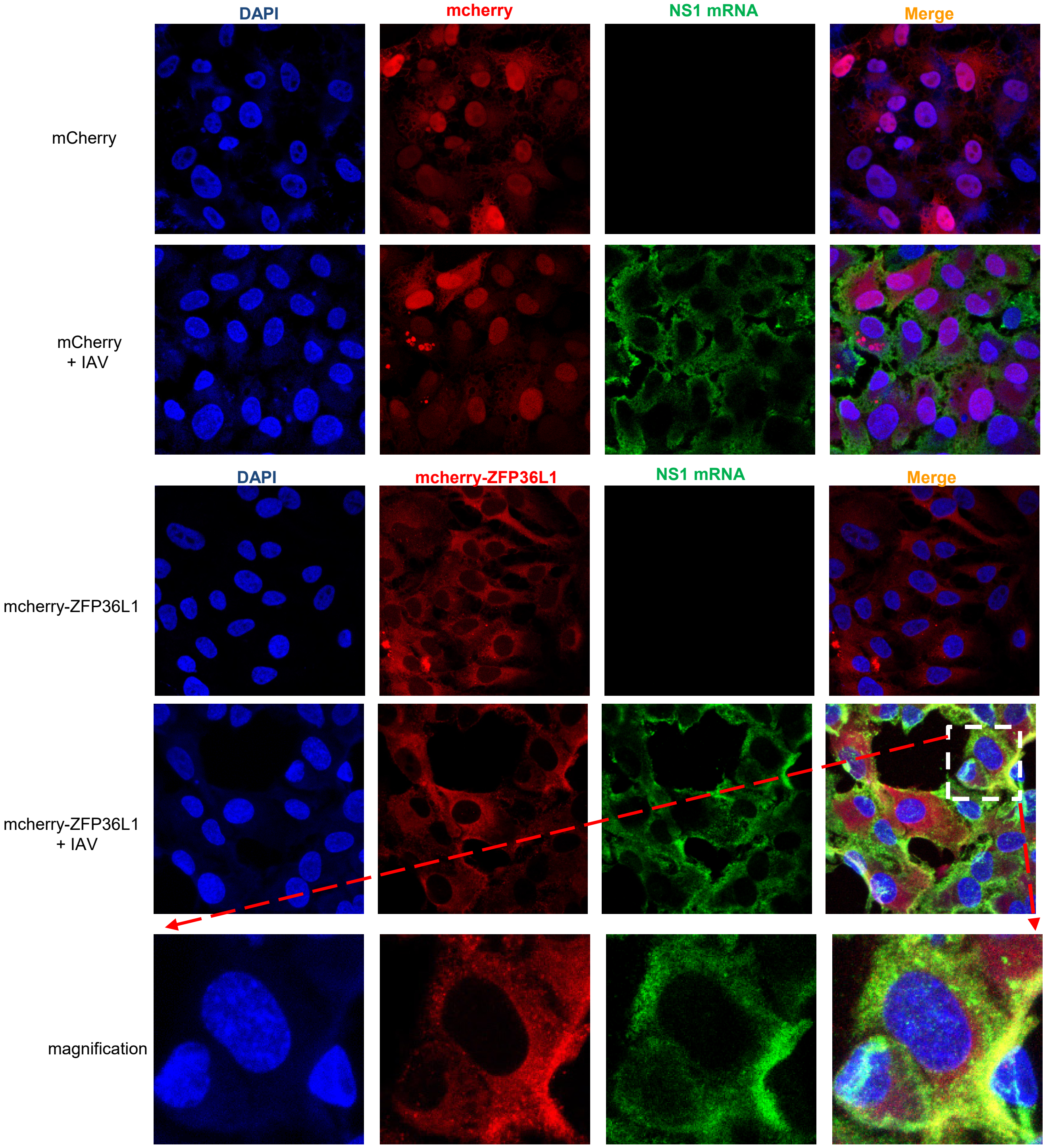

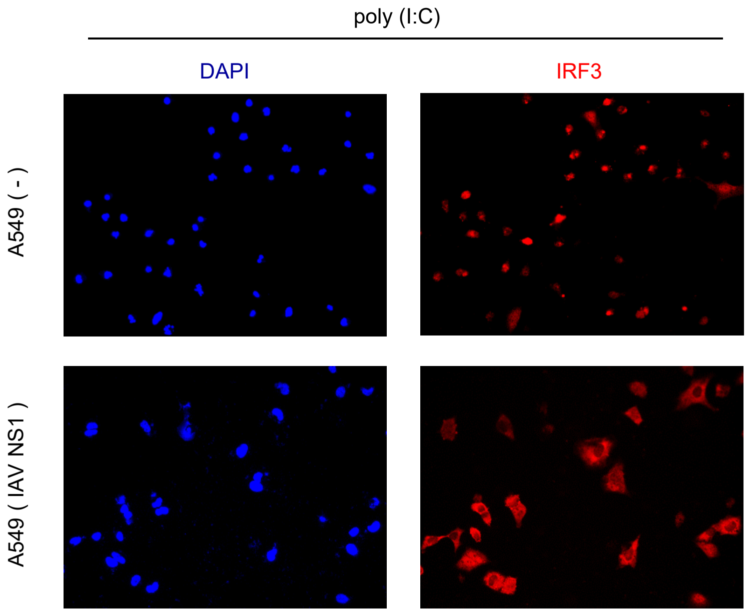
poly (I:C)
DAPI
IRF3
A549 (-)
A549 (IAV NS1)

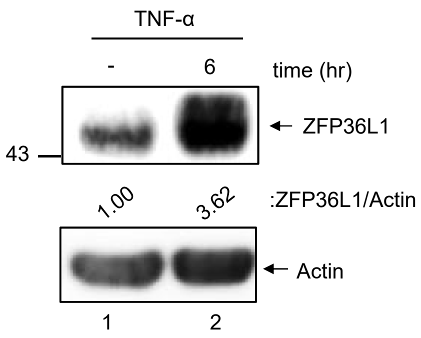
TNF-α
-
6
time (hr)
ZFP36L1
43
1.00
3.62
:ZFP36L1/Actin
Actin
1
2

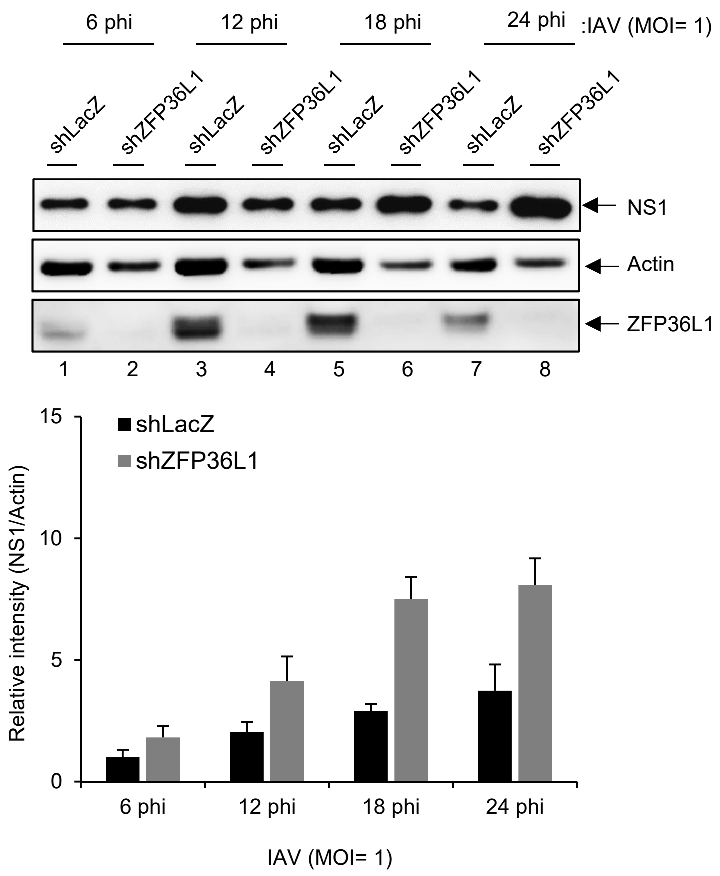
6 phi
12 phi
18 phi
24 phi
:IAV (MOI= 1)
shLacZ
shZFP36L1
shLacZ
shZFP36L1
shLacZ
shZFP36L1
shLacZ
shZFP36L1
NS1
Actin
ZFP36L1
1
2
3
4
5
6
7
8
15
10
5
0
shLacZ
shZFP36L1
Relative intensity (NS1/Actin)
6 phi
12 phi
18 phi
24 phi
IAV (MOI= 1)

A.

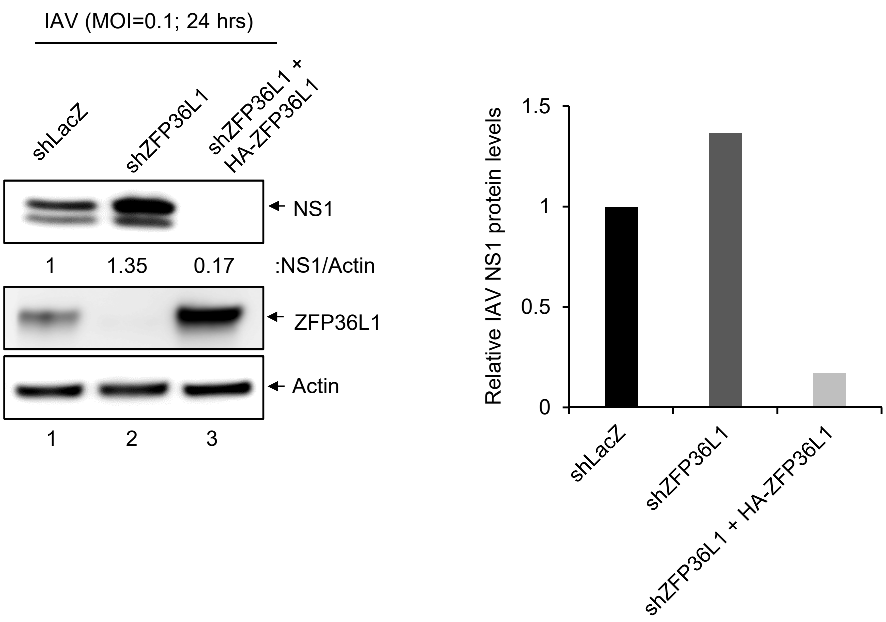
IAV (MOI=0.1; 24 hrs)
shLacZ
shZFP36L1
shZFP36L1 + HA-ZFP36L1
NS1
1
1.35
0.17
:NS1/Actin
ZFP36L1
Actin
1
2
3
Relative IAV NS1 protein levels
1.5
1
0.5
0
shLacZ
shZFP36L1
shZFP36L1 + HA-ZFP36L1

B.

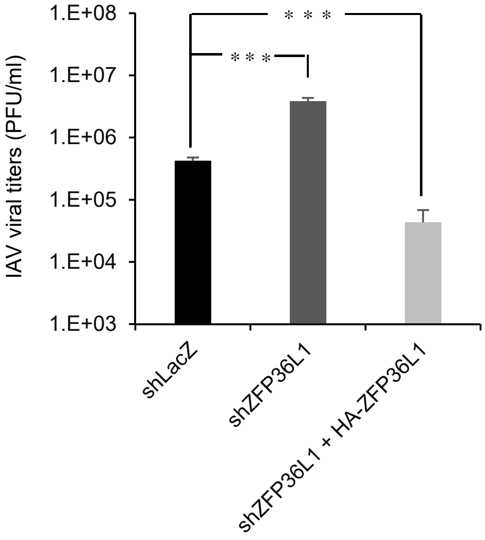
IAV viral titers (PFU/ml)
1.E+08
1.E+07
1.E+06
1.E+05
1.E+04
1.E+03
***
***
shLacZ
shZFP36L1
shZFP36L1 + HA-ZFP36L1

A.

NS1 mRNA

AGCAAAAGCAGGGUGACAAAGACAUAAUGGAUCCAAACACUGUGUCAAGCUUUCAGGUAGAUUGCUUUCUUUGGCAUGUC
CGCAAAAGAGUUGCAGACCAAGAACUAGGUUGAUGCCCCAUUCCUUGAUCGGCUUCGCCGAGAUCAGAAGUCCCUAAGAG
GAAGAGGCAGCACUCUCGGUCUGGACAUCGAAACAGCCACCCGUGCUGGAAAGCAAAUAGUGGAGCGGAUUCUGAAGGAA
GAAUCUGAUGAGGCACUCAAAAUGACCAUGGCCUCUGUACCUGCAUCGCGCUACCUAACUGACAUGACUCUUGAGGAAAU
GUCAAGGCACUGGUUCAUGCUCAUGCCCAAGCAGAAAGUGGCAGGCCCUCUUUGUAUCAGAAUGGACCAGGCGAUCAUGG
AUAAGAACAUCAUACUGAAAGCGAACUUCAGUGUGAUUUUUGACCGGCUGGAGACUCUAAUAUUACUAAGGGCCUUCACCG
AAGAGGGGACAAUUGUUGGCGAAAUUUCACCACUGCCCUCUCUUCCAGGACAUACUGAUGAGGAUGUCAAAAAUGCAGUU
GGGGUCCUCAUCGGAGGACUUGAAUGGAAUAAUAACACAGUUCGAGUCUCUGAAACUCUACAGAGAUUCGCUUGGAGAAG
CAGUAAUGAGAAUGGGAGACCUCCACUCACUCCAAAACAGAAACGGAAAAUGGCGGGAACAAUUAGGUCAGAAGUUUGAAG
AAAUAAGAUGGUUGAUUGAAGAAGUGAGACACAGACUGAAGAUAACAGAGAAUAGUUUUGAGCAAAUAACAUUUAUGCAAG
CCUUACAACUAUUGCUUGAAGUGGAGCAAGAGAUAAGAACUUUCUCGUUUCAGCUUAUUUAAUAAUAAAAAACACCCUUGU
UUCUACU

ORF   AUUUA pentamer for ZFP36L1 binding site

ARE1 mutant: AACAUUUAUGC → AACAGGGAUGC
ARE2 mutant: CUUAUUUAAUA → CUUAGGGAAUA

B.

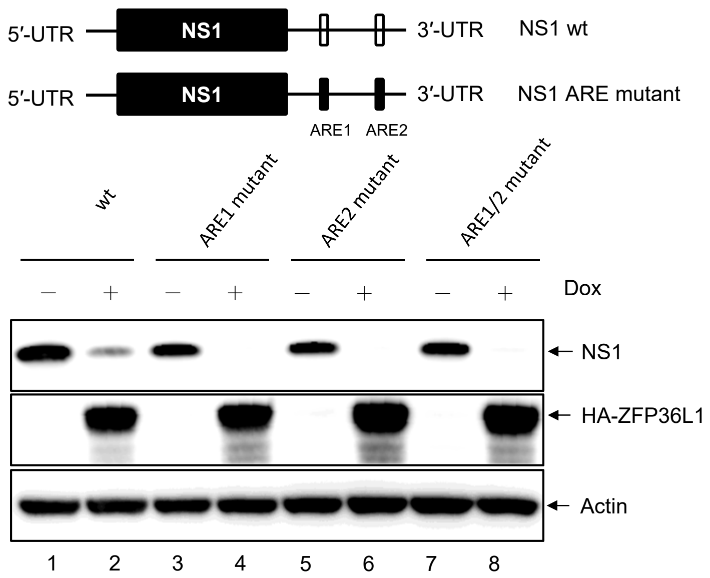

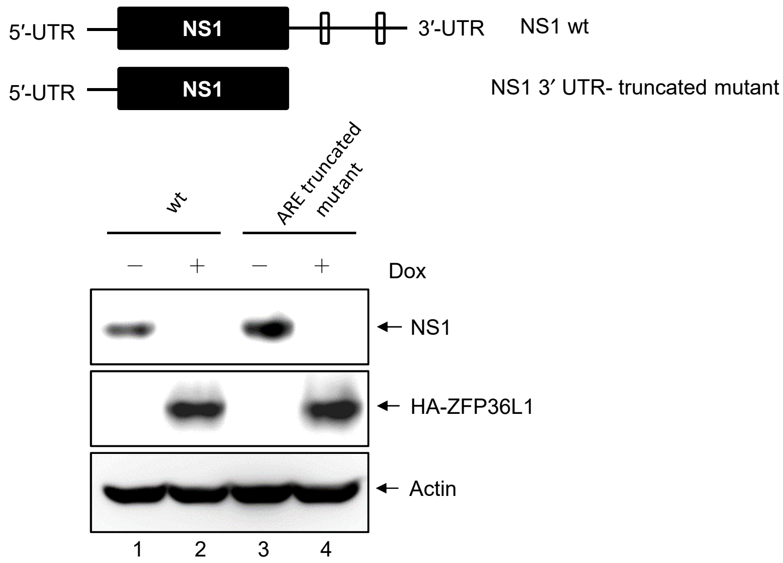

SUPPLEMENTARY FIGURE LEGEND

sFigure 1. A549 cells were transduced with the lentiviral vector (multiplicity of infection [MOI] = 2) expressing HA-ZFP36L1 or control EGFP for 72 h, then infected with IAV (MOI=1) for 6 h. Western blot analysis of IAV proteins (ribonucleoproteins: PB1, PB2, PA and NP; structural proteins: HA, NA, M1 and M2; nonstructural proteins: NS1 and NS2), HA-tagged ZFP36L1, and actin as a loading control. The relative quantification of IAV proteins normalized by actin was quantified by ImageJ software.

sFigure 2. T-REx-293 cells overexpressing ZFP36L1 induced with or without Dox (1 μg/ml) for 16 h. Cells were transfected with plasmids expressing IAV HA, M1, NS1 or NP. Western blot analysis of protein levels **(A)** and RT-PCR analysis of mRNA levels of IAV HA M1, NS1 and NP and actin as a loading control. **(B)** Quantitative real-time RT-PCR analysis of mRNA levels of IAV M1, NS1 and HA normalized to that of GAPDH. Data are mean ± SD of three independent experiments. NS: not significant.

sFigure 3. A549 cells were transduced with shRNA targeting control LacZ (shLacZ) or CNOT6 (shCNOT6). A549-shLacZ and -shCNOT6 cells were infected with IAV (MOI=1) for 6 h. Western blot analysis of protein levels of indicated IAV proteins, HA-tagged ZFP36L1, CNOT6, and actin as a loading control. The relative quantification of IAV proteins normalized by actin was quantified by ImageJ software.

sFigure 4. A549 cells were transduced with shRNA targeting control LacZ (shLacZ), XRN1 (shXRN1) or EXOSC5 (shEXOSC5). A549-shLacZ, -shXRN1 or -shEXOSC5 cells were infected with IAV (MOI=1) for 6 h. Western blot analysis of protein level of indicated IAV proteins, XRN1 **(A)**, EXOSC5 **(B)**, HA-tagged ZFP36L1 and actin as a loading control.

sFigure 5. T-REx-293 cells overexpressing ZFP36L1 with or without MG132 (0.5 μM) were transfected with plasmid expressing IAV NS1 for 24 h. Western blot analysis of protein levels of indicated IAV proteins NS1, M1, HA-tagged ZFP36L1 and actin as a loading control.

sFigure 6. A549 cells were transduced with the lentiviral vector (multiplicity of infection [MOI] = 2) expressing mcherry fused withZFP36L1 (mcherry-ZFP36L1; red) or control mcherry (red) for 72 h, then infected with IAV (MOI=1) for 6 h. Cells were fixed and permeabilized for RNA *in situ* hybridization (RNA ISH) and confocal laser scanning microscopy. RNA ISH was performed using the RNAscope Fluorescent Muiltplex Assay (Advanced Cell Diagnostics) according to the manufacturer's instructions. IAV NS1 mRNA were detected using a NS1 mRNA-specific probe (Advanced Cell Diagnostics) with sequential hybridization of amplifiers and FITC-labeled probe (green). The nuclei were stained with 4',6'-diamidino-2-phenylindole (DAPI; blue). Images were acquired using the Zeiss LSM 710 confocal microscope.

sFigure 7. A549 cells stably expressing IAV NS1 protein by lentivirus transduction were transfected with poly(I:C) (0.5 μg) for 24 h. Immunofluorescence assay of IRF3 protein (red)- and 4′,6'-diamidino-2-phenylindole (DAPI; blue)-stained cells.

sFigure 8. A549 cells were treated without or with TNF-α (20 ng/ml) for 6 h, then cell lysates were harvested for western blot analysis of protein levels of ZFP36L1 and actin as a loading control. The relative quantification of ZFP36L1 protein normalized by actin was quantified by ImageJ software.

sFigure 9. A549-shLacZ and -shZFP36L1 cells were infected with IAV (MOI=1) for the indicated times. Western blot analysis of protein level of IAV NS1, ZFP36L1 and actin as a loading control. The relative quantification of IAV proteins normalized by actin was quantified by ImageJ software.

sFigure 10. A549-shLacZ, -shZFP36L1, or -shZFP36L1 plus transduction with lentiviral vector expressing HA-tagged ZFP36L1 cells were infected with IAV (MOI = 0.1) for 24 h. **(A)** Western blot analysis of protein levels of IAV NS1, ZFP36L1 and actin as a loading control. The relative quantification of IAV proteins normalized by actin was quantified by ImageJ software. **(B)** Plaque-formation assay of infectious IAV titers (PFU/ml) in MDCK cell culture supernatants. Titers of the indicated groups were compared by two-tailed Student t test. Data are mean ± SD of three independent experiments. ***$P<0.001$.

sFigure 11. (A) Schematic representation of nucleotide sequence of IAV mRNA transcript with open-reading flame (ORF; red letters) and AU-rich region (ARE; green letters). **(B)** Human T-REx-293 cells with or without ZFP36L1 overexpression were transfected with plasmids expressing IAV NS1 *wt*, NS1-ARE1 mutant, NS1-ARE2 mutant, NS1-ARE1/2 mutant or NS1 3′ UTR-truncated mutant, respectively. Western blot analysis of f protein level of IAV NS1, HA-ZFP36L1 and actin as a loading control.
